# Clinical coding of long COVID in primary care 2020–2023 in a cohort of 19 million adults: an OpenSAFELY analysis

**DOI:** 10.1016/j.eclinm.2024.102638

**Published:** 2024-05-17

**Authors:** Alasdair D. Henderson, Ben FC. Butler-Cole, John Tazare, Laurie A. Tomlinson, Michael Marks, Mark Jit, Andrew Briggs, Liang-Yu Lin, Oliver Carlile, Chris Bates, John Parry, Sebastian CJ. Bacon, Iain Dillingham, William A. Dennison, Ruth E. Costello, Yinghui Wei, Alex J. Walker, William Hulme, Ben Goldacre, Amir Mehrkar, Brian MacKenna, The OpenSAFELY Collaborative, Alex Walker, Amelia Green, Amir Mehrkar, Andrea Schaffer, Andrew Brown, Ben Goldacre, Ben Butler-Cole, Brian MacKenna, Caroline Morton, Caroline Walters, Catherine Stables, Christine Cunningham, Christopher Wood, Colm Andrews, David Evans, George Hickman, Helen Curtis, Henry Drysdale, Iain Dillingham, Jessica Morley, Jon Massey, Linda Nab, Lisa Hopcroft, Louis Fisher, Lucy Bridges, Milan Wiedemann, Nicholas DeVito, Orla Macdonald, Peter Inglesby, Rebecca Smith, Richard Croker, Robin Park, Rose Higgins, Sebastian Bacon, Simon Davy, Steven Maude, Thomas O'Dwyer, Tom Ward, Victoria Speed, William Hulme, Liam Hart, Pete Stokes, Krishnan Bhaskaran, Ruth Costello, Thomas Cowling, Ian Douglas, Rosalind Eggo, Stephen Evans, Harriet Forbes, Richard Grieve, Daniel Grint, Emily Herrett, Sinead Langan, Viyaasan Mahalingasivam, Kathryn Mansfield, Rohini Mathur, Helen McDonald, Edward Parker, Christopher Rentsch, Anna Schultze, Liam Smeeth, John Tazare, Laurie Tomlinson, Jemma Walker, Elizabeth Williamson, Kevin Wing, Angel Wong, Bang Zheng, Christopher Bates, Jonathan Cockburn, John Parry, Frank Hester, Sam Harper, Shaun O'Hanlon, Alex Eavis, Richard Jarvis, Dima Avramov, Paul Griffiths, Aaron Fowles, Nasreen Parkes, Rafael Perera, David Harrison, Kamlesh Khunti, Jonathan Sterne, Jennifer Quint, Emily Herrett, Rosalind M. Eggo

**Affiliations:** a London School of Hygiene and Tropical Medicine, Keppel Street, London WC1E 7HT, UK; b Bennett Institute for Applied Data Science, Nuffield Department of Primary Care Health Sciences, University of Oxford, OX2 6GG, UK; c TPP, TPP House, 129 Low Lane, Horsforth, Leeds LS18 5PX, UK; d Patient and Public Involvement Steering Committee, London, UK; e Centre for Mathematical Sciences, School of Engineering, Computing and Mathematics, University of Plymouth, Plymouth, UK

**Keywords:** Long COVID, Vaccination, Descriptive cohort

## Abstract

**Background:**

Long COVID is the patient-coined term for the persistent symptoms of COVID-19 illness for weeks, months or years following the acute infection. There is a large burden of long COVID globally from self-reported data, but the epidemiology, causes and treatments remain poorly understood. Primary care is used to help identify and treat patients with long COVID and therefore Electronic Health Records (EHRs) of past COVID-19 patients could be used to help fill these knowledge gaps. We aimed to describe the incidence and differences in demographic and clinical characteristics in recorded long COVID in primary care records in England.

**Methods:**

With the approval of NHS England we used routine clinical data from over 19 million adults in England linked to SARS-COV-2 test result, hospitalisation and vaccination data to describe trends in the recording of 16 clinical codes related to long COVID between November 2020 and January 2023. Using OpenSAFELY, we calculated rates per 100,000 person-years and plotted how these changed over time. We compared crude and adjusted (for age, sex, 9 NHS regions of England, and the dominant variant circulating) rates of recorded long COVID in patient records between different key demographic and vaccination characteristics using negative binomial models.

**Findings:**

We identified a total of 55,465 people recorded to have long COVID over the study period, which included 20,025 diagnoses codes and 35,440 codes for further assessment. The incidence of new long COVID records increased steadily over 2021, and declined over 2022. The overall rate per 100,000 person-years was 177.5 cases in women (95% CI: 175.5–179) and 100.5 in men (99.5–102). The majority of those with a long COVID record did not have a recorded positive SARS-COV-2 test 12 or more weeks before the long COVID record.

**Interpretation:**

In this descriptive study, EHR recorded long COVID was very low between 2020 and 2023, and incident records of long COVID declined over 2022. Using EHR diagnostic or referral codes unfortunately has major limitations in identifying and ascertaining true cases and timing of long COVID.

**Funding:**

This research was supported by the 10.13039/501100000272National Institute for Health and Care Research (NIHR) (OpenPROMPT: COV-LT2-0073).

**Research in context**

**Evidence before this study**

We searched PubMed for research published between 1st January 2020 and 1st November 2023, published in English, for the Title/Abstract terms (Post COVID-19 Condition OR PCC OR post-acute-covid-19 OR PASC OR long covid) AND (Electronic Health Records OR routinely collected health∗) AND (prevalence OR descriptive OR describe). Of the 13 studies identified, 2 were irrelevant and misclassified, 1 was a protocol without results, 4 described coded symptoms before or after COVID-19 infection, 2 defined bespoke long COVID definitions through coded symptoms, 3 used ICD-10 code to define long COVID and 1 used the same long COVID definition as our study, but only included data up to May 2021.

**Added value of this study**

We analysed the healthcare records of 19 million adults in England using the OpenSAFELY platform to describe the dynamics of long COVID coding in primary care. We found 55,465 records of long COVID between November 2020 and January 2023, many fewer than the number estimated by the Office for National Statistics. We also describe which subgroups of the population had higher incidence of recorded long COVID, and that limiting case definitions to those with evidence of COVID-19 infection severely restricts the potential size of long COVID cohorts.

**Implications of all the available evidence**

Electronic health records are a valuable resource to study the long-term effects of COVID-19, however the variety of different definitions being used in research makes it hard to generalise between studies. There are differences in use of codes through time which affects definition of long COVID and may have implications for future research. We have carefully demonstrated the strengths and limitations of using recorded long COVID in primary care to define long COVID. Our results suggest that harmonisation of definitions of long COVID between studies is needed.

## Introduction

Some people experience prolonged symptoms for weeks or months following acute SARS-COV-2 infection. This sequelae is known as long COVID, which is probably best currently conceptualised not as a single disease entity but as a classification designed to include all individuals who develop persistent symptoms following acute SARS-CoV-2 infection. This classification likely represents multiple underlying syndromes including cardiovascular, thrombotic and cerebrovascular disease, myalgic encephalomyelitis/chronic fatigue syndrome and dysautonomia,^1^^,^^2^ each with distinct pathophysiologies and prognoses^3^, ^4^, ^5^, ^6^, ^7^ and in some individuals these symptoms can be long-lasting and severe.^8^, ^9^, ^10^, ^11^ The heterogeneity within the classification contributes to inconsistent definitions of long COVID across studies with resulting wide variation in estimated prevalence^12^, ^13^, ^14^, ^15^, ^16^ and risk of developing long COVID following SARS-COV-2 infection.^17^, ^18^, ^19^, ^20^, ^21^

Given this uncertainty, more research of the causes and consequences of long COVID is necessary.^22^ Electronic health records (EHRs) are a possible data source for this research and they have become critical in healthcare research,^23^, ^24^, ^25^, ^26^ therefore careful analysis of EHRs could present an opportunity to better understand long COVID.^27^, ^28^, ^29^, ^30^, ^31^ However, common problems with EHR data include diagnostic accuracy, inconsistent coding, missing data and ascertainment bias.^24^^,^^32^^,^^33^ In the UK, diagnostic and referral codes for long COVID have been available for General Practitioners (GPs) in the UK since November 2020, along with guidelines on use of these codes from the National Institute for Clinical Excellence (NICE guideline [NG188]).

We have previously summarised the early clinical coding of long COVID up to May 2021 and shown very low recording of long COVID.^27^ However, since then, different SARS-COV-2 variants have emerged and many COVID-19 vaccines administered, and there have likely been changes in coding practices. It is vital to understand any potential differences in coding before we can use EHRs to answer more complex research questions about long COVID. We therefore set out to comprehensively describe the incidence of GP-recorded long COVID in adults in England, and stratified by demographic and clinical characteristics using OpenSAFELY.

## Methods

### Data source

We used a database of 19 million adults in England, whose primary care records are managed by the GP software provider TPP SystmOne. This is the software platform used by approximately 40% of GP practices across England^34^ where information on diagnoses, referrals, prescription, and other health data are entered. We accessed these data through the OpenSAFELY platform, where all data were linked, stored and analysed securely (https://opensafely.org/). OpenSAFELY is a secure analytics platform for research using patient health records. The platform uses a new model for enhanced security and timely access to data: data stays in the secure environment in which it is stored for individual care. Data, including coded diagnoses, medications and physiological parameters, are pseudonymised. No free text data are included. The following linked data were also used for this study: patient-level COVID-19 vaccination status via the National Immunisation Management System (NIMS); in-patient hospital spell records via NHS Digital’s Hospital Episode Statistics (HES); national coronavirus testing records via the Second Generation Surveillance System (SGSS); Detailed pseudonymised patient data are potentially re-identifiable and therefore not shared.

The OpenSAFELY platform team has developed a publicly available website https://opensafely.org/ which describes the platform in language suitable for a lay audience. This specific study design was developed with input from our patient and public advisory panel through regular meetings, and following feedback from two large workshops to inform the research design, develop understanding of the lived experience of long COVID, and to improve communication of results to patients and the public, hosted in January and September 2023.^35^^,^^36^

### Study population

We included all individuals aged 18–100 years and registered (temporary registrations were excluded) with a general practice that uses TPP SystmOne software on or after 1 November 2020, the date that long COVID Systemized Nomenclature of Medicine (SNOMED) codes became available. SNOMED codes are a dictionary of computer-readable codes relating to clinical terms.^37^ Participants were followed up from the beginning of their registration plus 90 days to account for onboarding of EHR records after registering at a new practice. Participants were then followed until the earliest of: EHR record of long COVID; end of registration with the same general practice; death; or 31st January 2023 (Supplementary Figs. S1–S3^38^).

### Outcomes

The primary outcome was the first GP record of long COVID defined by 15 SNOMED codes, as used previously^27^^,^^39^, ^40^, ^41^ which are split between two groups of codes: diagnosis and referral (Table 1). Our definition of recorded long COVID was therefore not exclusively clinical diagnoses of long COVID. Records were searched for a diagnosis code first (Any long COVID diagnosis code), if no code existed then we searched for a referral code (Any long COVID code). If neither code type existed then the individual was classified as not having long COVID.

**Table 1:** List of SNOMED-CT codes used to identify long COVID in the EHR record.

SNOMED CT code	Description
Diagnosis codes	
1325161000000102	Post-COVID-19 syndrome
1325181000000106	Ongoing symptomatic disease caused by severe acute respiratory syndrome coronavirus 2
Referral codes	
1325021000000106	Signposting to Your COVID Recovery
1325031000000108	Referral to post-COVID assessment clinic
1325041000000104	Referral to Your COVID Recovery rehabilitation platform
1325051000000101	Newcastle post-COVID syndrome Follow-up Screening Questionnaire
1325061000000103	Assessment using Newcastle post-COVID syndrome Follow-up Screening Questionnaire
1325071000000105	COVID-19 Yorkshire Rehabilitation Screening tool
1325081000000107	Assessment using COVID-19 Yorkshire Rehabilitation Screening tool
1325091000000109	Post-COVID-19 Functional Status Scale patient self-report
1325101000000101	Assessment using Post-COVID-19 Functional Status Scale patient self-report
1325121000000105	Post-COVID-19 Functional Status Scale patient self-report final scale grade
1325131000000107	Post-COVID-19 Functional Status Scale structured interview final scale grade
1325141000000103	Assessment using Post-COVID-19 Functional Status Scale structured interview
1325151000000100	Post-COVID-19 Functional Status Scale structured interview

We also included a “control outcome”, hospitalisation with COVID-19 (Supplementary Methods), for which we have more evidence for the expected negative association with SARS-COV-2 vaccination,^38^^,^^39^ while this association is more uncertain for long COVID.^42^^,^^43^ To account for the gap between SARS-CoV-2 infection and the recording of long COVID, we only analysed COVID-19 test results and hospitalisations >12 weeks before the end of follow up. Data on SARS-CoV-2 test results were available from the SGSS.

### Stratifiers

COVID-19 vaccination status was the only time-updated covariate and was categorised in two ways: i) follow-up was divided by the number of vaccine doses received (0, 1, 2, 3+); ii) participants were categorised as having received an mRNA-based vaccine (Pfizer (Comirnarty), Moderna (Spikevax)) for their first immunisation, or a non-mRNA vaccine. Only vaccine doses greater than 14 weeks before the end of follow up were included to account for the gap between immunisation and protection (2 weeks) and development of long COVID symptoms (12 weeks).

All other covariates were defined at baseline, the start of a valid registration with a GP in the study period. Age (18–29, 30–39, 40–49, 50–59, 60–69, 70+) and sex were defined on the registration record. NHS region (9 regions in England), and index of multiple deprivation (IMD) quintiles were based on the address of each participant. Ethnicity (categorised as white, Black, South Asian, mixed, Other),^44^ and those at high-risk of complications from COVID-19^45^ were assessed from primary care records. The presence of fifteen chronic comorbidities identified as increasing the risk of severe COVID-19 disease from previous research^15^ were defined using primary care records at baseline and categorised (0, 1, 2+), the full list of comorbidities is available in the Supplementary Methods. Finally, we defined two binary “probable shielding” variables. Shielding was recommended for those at risk of complications from SARS-COV-2 infection to reduce the chance of infection. One variable was defined for those at “high-risk” of complications from SARS-CoV-2 infection, and one for those at low/moderate-risk. Both shielding variables were based on the presence of the corresponding SNOMED code.^43^ We use the term “probable” shielding as there is uncertainty about whether presence of a code was correlated with that individual actually following shielding guidelines.

### Statistics

We estimated the crude rate of long COVID per 100,000 person years and 95% confidence intervals for each level of each stratifier listed (Supplementary Methods). All counts for presentation were rounded to the nearest 5 and counts lower than 10 were redacted to ensure results are non-disclosive.

To compare rates between levels of each stratifier while partially adjusting for confounding, we developed negative binomial models for age category, sex and vaccination. All models were adjusted for age, sex, NHS region and dominant SARS-CoV-2 variant period (wildtype/alpha, 1 November 2020–16 May 2021; Delta, 16 May 2021–1 December 2021; Omicron, 1 December 2021–31 January 2023^46^) to estimate rate ratios.

We graphically presented the monthly incidence of long COVID recording by specific SNOMED codes, and SARS-COV-2 test positive results between November 2020 and January 2023. We also described the reported SARS-CoV-2 history of people with EHR records of long COVID in a Sankey diagram, from SARS-CoV-2 test status, to COVID-19 hospitalisation to EHR recorded long COVID. We then compared the characteristics of those with and without a recorded SARS-CoV-2 positive test before their record of long COVID.

We expanded the negative binomial models further by running separate models for each of the three variant periods to analyse the consistency of these associations across the pandemic. We also conducted a sensitivity analysis of our vaccine definition by including vaccinations >14 weeks before end of follow-up (main analysis), to results from >16 weeks to >26 weeks.

Finally, we used the Secondary Cohort and calculated the percentage of people with either 0, 1, 2, or 3+ vaccine doses at the end of the study, stratified by whether they had a long COVID record previously and by age group.

### Ethics

This research is part of the OpenPROMPT study “Quality-of-life in patients with long COVID: harnessing the scale of big data to quantify the health and economic costs” which has ethical approval from HRA and Health and Care Research Wales (HCRW) (IRAS project ID 304354). The Study Coordination Centre has obtained approval from the LSHTM Research Ethics Committee (ref 28030), as well as a favourable opinion from the South Central–Berkshire B Research Ethics Committee (ref 22/SC/0198). Full ethical approval details are available online (Supplementary Methods). The OpenSAFELY platform was established using legal powers that set aside the requirement for patient consent (Appendix p 2).

### Role of funding source

This work is independent research funded by the National Institute for Health and Care Research (NIHR) [OpenPROMPT: COV-LT2-0073]. The views expressed in this publication are those of the author(s) and not necessarily those of NIHR or The Department of Health and Social Care. In addition, this research used data assets made available as part of the Data and Connectivity National Core Study, led by Health Data Research UK in partnership with the Office for National Statistics and funded by UK Research and Innovation (grant ref MC_PC_20058). In addition, the OpenSAFELY Platform is supported by grants from the Wellcome Trust (222097/Z/20/Z); MRC (MR/V015737/1, MC_PC-20059, MR/W016729/1); NIHR (NIHR135559, COV-LT2-0073), and Health Data Research UK (HDRUK2021.000, 2021.0157). YW was supported by a UKRI MRC Fellowship (MC/W021358/1) and Health Data Research UK, and received funding from the UKRI EPSRC Impact Acceleration Account (EP/X525789/1).

The views expressed are those of the authors and not necessarily those of the NIHR, NHS England, UK Health Security Agency (UKHSA) or the Department of Health and Social Care. Funders had no role in the study design, collection, analysis, and interpretation of data; in the writing of the report; and in the decision to submit the article for publication.

## Results

### Variation in incidence of long COVID recording in England

We analysed data from 19,462,260 adults in England between November 2020 and January 2023 with a median follow up time of 2.2 years. There was an even split of men and women, and 70% of the cohort were recorded as white ethnicity. Most of the cohort lived in the East Midlands (17%), East (23%), South West (14%) and Yorkshire & the Humber (14%) reflecting where SystmOne is used. Over a third of the cohort had at least one chronic comorbidity (Table 2).

**Table 2:** Baseline cohort characteristics.

Variable	Level	Cohort summary
Total		19,462,080
Follow-up start (year)	2020	17,824,820 (91.6%)
	2021	884,790 (4.5%)
	2022	675,120 (3.5%)
	2023	77,530 (0.4%)
Sex	Male	9,720,385 (49.9%)
	Female	9,741,875 (50.1%)
Age (IQR)		48 (33–63)
Age category	18–29	4,078,305 (21%)
	30–39	3,450,885 (17.7%)
	40–49	3,129,670 (16.1%)
	50–59	3,271,525 (16.8%)
	60–69	2,527,420 (13%)
	70+	3,004,460 (15.4%)
Ethnicity	White	13,586,060 (69.8%)
	Mixed	234,525 (1.2%)
	South Asian	1,356,505 (7%)
	Black	470,675 (2.4%)
	Other	514,690 (2.6%)
	(Missing)	3,299,805 (17%)
Region	London	1,559,650 (8%)
	East Midlands	3,298,290 (16.9%)
	East	4,403,440 (22.6%)
	North East	904,835 (4.6%)
	North West	1,702,730 (8.7%)
	South East	1,288,965 (6.6%)
	South West	2,791,195 (14.3%)
	West Midlands	789,260 (4.1%)
	Yorkshire and The Humber	2,709,565 (13.9%)
	(Missing)	14,340 (0.1%)
IMD (quintile)	1 (most deprived)	3,508,485 (18%)
	2	3,677,465 (18.9%)
	3	3,990,465 (20.5%)
	4	3,761,210 (19.3%)
	5 (least deprived)	3,499,345 (18%)
	(Missing)	1,025,285 (5.3%)
Comorbidities	0	12,441,695 (63.9%)
	1	4,966,470 (25.5%)
	2+	2,054,095 (10.6%)
Probably shielding (high risk group)		957,765 (4.9%)
Probably shielding (Low/moderate risk group)		463,750 (2.4%)

Figures shown are n (%) for binary and categorical variables, median (25%–75% percentile) for continuous variables.

**Fig. 1:**
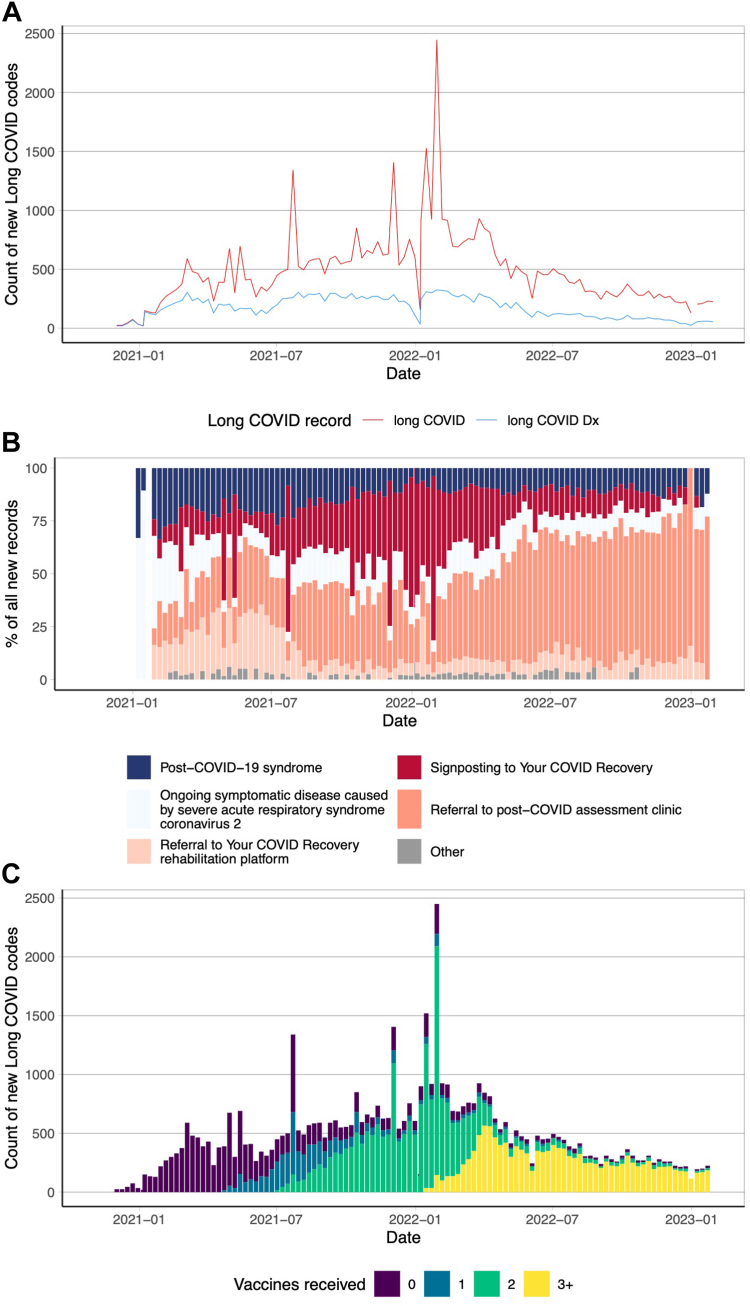
Dynamics of long COVID recording in EHRs. A: Weekly count of long COVID codes (any long COVID code, red; of which were diagnosis codes, blue). Records were searched for a diagnosis code first (Any long COVID diagnosis code), if no code existed then we searched for a referral code (Any long COVID code). If neither code type existed then the individual was classified as not having long COVID. B: Weekly proportion of the 5 most common long COVID codes amongst all new long COVID codes recorded that week. C: Weekly count of all long COVID codes stratified by the number of vaccine doses received ≥14 weeks prior to the long COVID code.

We identified 55,465 individuals with recorded codes for long COVID, 20,025 of which were diagnosis codes, with the remaining 35,440 referral codes (Supplementary Table S1). The number of newly recorded (incident) long COVID cases increased steadily over 2021, before peaking in January 2022 and declining steadily for the following 12 months (Fig. 1). The dynamics of which specific codes were used has changed over time. We used a hierarchical search to identify all long COVID diagnosis codes initially, and then search for referral codes if no diagnosis existed (Supplementary Methods). This means the diagnosis code was preferred even if there was an earlier referral code. Despite this hierarchy, there were more referral codes recorded in our study and this proportion increased over time. Since mid-2022, the majority of new records have been referrals to post-COVID assessment clinics (Fig. 1B). Initially, all long COVID records were recorded in unvaccinated individuals but over the course of the study period as people received SARS-COV-2 vaccinations more clinical codes for long COVID were recorded in people with 1 or more vaccinations (Fig. 1C), which is consistent when stratified by sex (Supplementary Fig. S4).

**Fig. 2:**
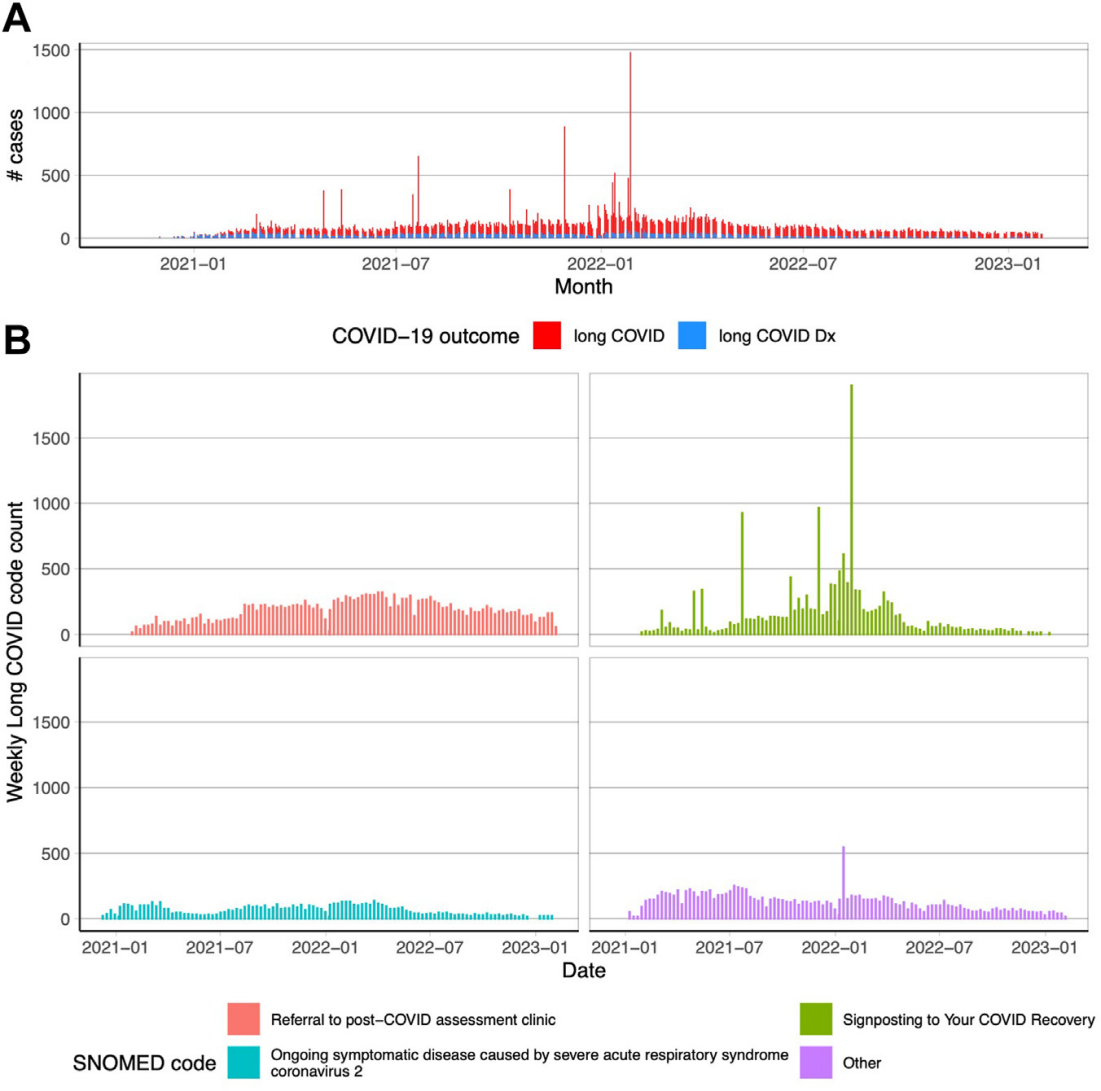
Primary care coding of long COVID codes over time. A: Daily counts of any long COVID code (red) and long COVID diagnoses only (blue). Records were searched for a diagnosis code first (Any long COVID diagnosis code), if no code existed then we searched for a referral code (Any long COVID code). If neither code type existed then the individual was classified as not having long COVID. B: Weekly counts of the three most common long COVID codes in primary care, and the remaining codes grouped as “other”. Counts less than 10 are suppressed.

The weekly pattern appeared to mask more marked variation in the recording of long COVID codes and we identified certain dates with large spikes (Fig. 2). The main cause of these outliers in the time series appeared to be the use of one SNOMED code (“Signposting to Your COVID Recovery”) with three notable spikes in July 2021, December 2021 and January 2022. The pattern of long COVID recording over time did peak at the same time as SARS-COV-2 infections at a national level, but did not reflect the decline in infections in early 2021 or the waves of infections in 2022 (Supplementary Fig. S5).

### Recorded long COVID rates vary between population groups

**Fig. 3:**
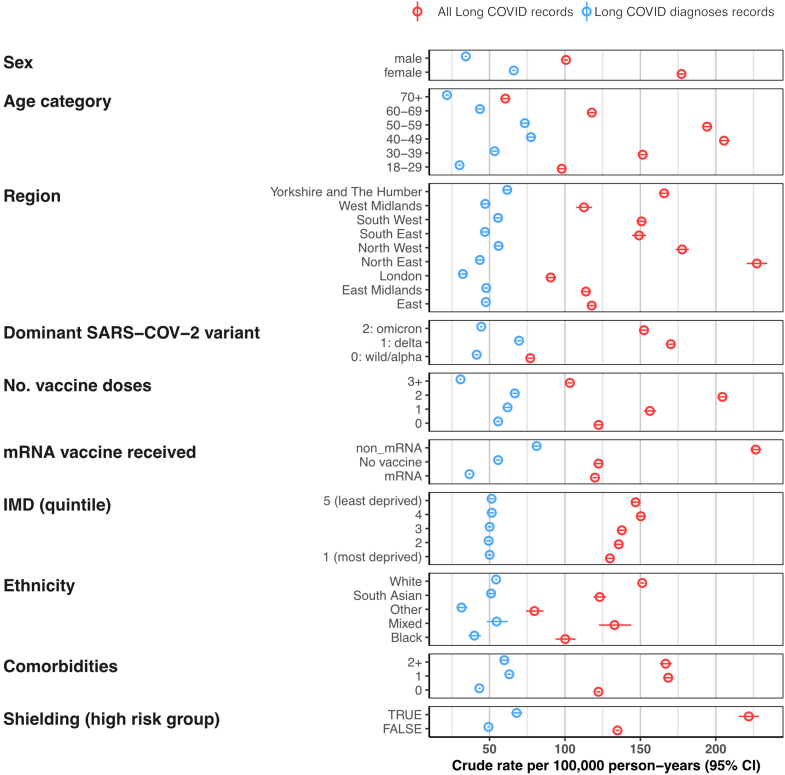
Rates of recorded long COVID in primary care records per 100,000 person-years. Rate of any long COVID code (red) and long COVID diagnoses only (blue). IMD: index of multiple deprivation. Records were searched for a diagnosis code first (Any long COVID diagnosis code), if no code existed then we searched for a referral code (Any long COVID code). If neither code type existed then the individual was classified as not having long COVID.

Crude rates of long COVID coding were highest for women, ages 40–60, white ethnicity, those with at least one comorbidity and people who were shielding because they were at high-risk of complications from COVID-19 (Fig. 3). The crude rate of long COVID records were lowest in those with 3+ vaccine doses, and were lower for those who received an mRNA-based vaccine as their first dose. However, the raw rate of long COVID codes was higher in those with one or two doses of the vaccine (Fig. 1C). Finally, some patterns in the crude rates of EHR recorded long COVID were dependent on whether referral codes are included in the definition. Notably, we found that long COVID codes were more likely to occur in people living in less deprived areas, however this association did not hold when long COVID diagnosis codes only were analysed.

We conducted exploratory analysis of differences in long COVID rates by age, sex and vaccination status. These analyses show that long COVID rates were lowest for people with 3 or more vaccine doses (103.5 per 100,000 person-years; 95% CI: 101.5–105), although these results should not be interpreted causally (Supplementary text, Figs. S7–S10, and Tables S1 and S2).

### Differences in long COVID EHR recording routes and relation to SARS-CoV-2 testing

**Fig. 4:**
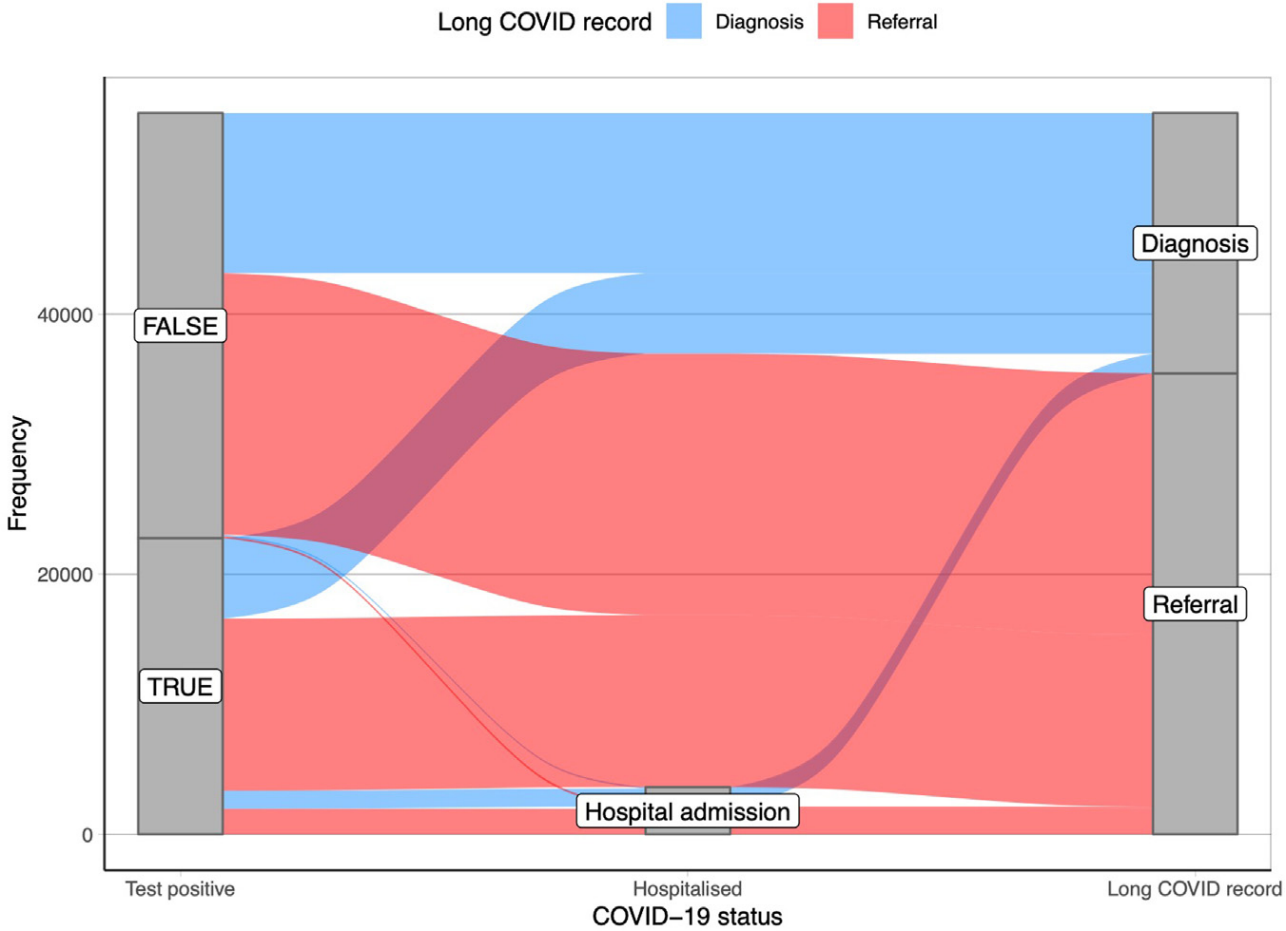
Sankey diagram of the transition from the presence of a SARS-COV-2 test to a COVID-19 hospitalisation to a first long COVID record in primary care for 55,465 participants with a long COVID record.

Finally, we investigated the pathways to a long COVID record. We examined the linked SARS-CoV-2 tests and COVID-19 hospitalisation data to calculate the proportion of the 55,465 people with a long COVID record that had previously recorded a positive SARS-CoV-2 test, and been hospitalised with COVID-19. We found that the majority of people with a long COVID record (59%) did not have a recorded positive test result ≥12 weeks before the long COVID record, and a small minority (6.5%) were hospitalised with COVID-19 (Fig. 4). There were systematic differences between those with and without a positive test amongst all participants with a long COVID record: those with a previous positive test result were more likely to be female, older, from a more deprived IMD quintile, vaccinated, and to have not been hospitalised with COVID-19 (Supplementary Table S3).

## Discussion

We analysed the health records of over 19 million adults in England and found very low rates of GP recorded long COVID diagnoses and referrals for long COVID care. We found that referral codes were increasingly common across 2021, but the rate of newly coded patients steadily declined over the year 2022. We found that the choice of long COVID codes used in EHR research to define a long COVID phenotype will have a notable impact on the number of outcomes and temporal dynamics. We do not know if referral or diagnosis codes indicate any difference in severity of symptoms, but there are demographic differences in who received each type of code. Therefore, future studies defining long COVID in these ways should be aware of the different populations represented by each type of code. We also described wide regional variation in long COVID coding and an increase in long COVID referrals in less deprived areas, which may be indicative of greater access to care in these areas.^47^

A major strength of our study is the size of the data available. We analysed data on 19 million people and we were able to safely analyse linked primary care, hospitalisation, and SARS-COV-2 test data due to the OpenSAFELY architecture.^48^ We used a previously defined codelist to identify those with long COVID and to facilitate easy comparisons from research conducted during the pandemic.^27^ Our analysis has also demonstrated issues with using EHR data for further more complex analyses of long COVID, for example target trial estimation of vaccine efficacy which has been done for other COVID-19 outcomes.^42^^,^^43^

The aim of EHR research is to accurately identify people with a health condition from their medical record. This is challenging and using a single code is often insufficient,^49^ and may lead to bias from misclassification.^50^^,^^51^ We have identified several areas where this may not be feasible for long COVID, which is why we have been explicit throughout the report that we are describing the coding of long COVID, rather than the true incidence of the condition. Our study suggests that it is likely many people who self-report long COVID in surveys will not have a record from their GP, or will have the diagnosis captured in the “free text” of their medical record so will not have been captured by our study.^52^^,^^53^ It is also possible that people with a code for long COVID in their GP records do not have the condition, especially those with a referral code as the referral may have concluded that they did not have long COVID. It is also likely that some people will recover from long COVID during this study period which we cannot capture with routine care records. The low incidence of these codes reduces the possibility of using multiple codes on non-consecutive days to define a more specific long COVID population as this would reduce sample sizes even further. Fundamentally, there is currently no gold standard to compare recorded long COVID with in order to assess the accuracy of these codes, however our research demonstrates evidence that research using these codes should proceed with caution.

Our study demonstrates limitations of using long COVID coding to determine the presence or absence of the condition, but these codes are even further limited in determining the timing of disease onset. There may be systematic differences between people’s propensity to visit their GP with continuing COVID-19 symptoms following an acute COVID-19 infection. There is an additional possible bias in the recording of positive test results because there may be differences in the rate of false test results over the pandemic,^52^^,^^53^ and because of potential systematic differences in the propensity to record test results between those who do and do not receive a record of long COVID in primary care. We have shown that long COVID codes may even be more closely aligned with acute infections rather than continued symptoms, as the peak of long COVID codes and peak of nationally reported COVID-19 infections overlap (Supplementary Fig. S5), where we would expect long COVID coding to peak 1–3 months after the peak of infections. These issues combined severely limit the possibility of causal inference using these data, as we demonstrated in the Appendix (Supplementary Text).

We only had access to practices using SystmOne software in this study, whereas previous work showed that rates of long COVID coding were higher in practices using EMIS software.^27^ We also assumed that rates were constant over time by using a negative binomial model. This does not allow for changes in the rate that may be directly influenced by the availability of SNOMED codes, changes in clinical guidelines or the availability of long COVID support services. These highlighted limitations demonstrate that findings from this study should not be generalised, in addition we observed incomplete data for key demographic variables, notably ethnicity (17% missing), so it is unclear what population so it is unclear how these results would generalise to the complete population, even if capturing long COVID records was more accurate.

Long COVID codes are rarely recorded in primary care compared to the estimated 2.1 million cases of long COVID self reported in the proactively sampled ONS community infection survey.^12^ If we assume a crude 10% of SARS-CoV-2 infections result in long COVID, as elsewhere,^14^ and with approximately 20 million recorded infections in England^46^ the number of recorded long COVID cases in primary care is an order of magnitude below the estimated incidence of long COVID in England given the number of SARS-CoV-2 infections. Our findings agree with previous work, that there are serious limitations with simply using EHR records as a measure of long COVID^30^^,^^54^, ^55^, ^56^ and alternative approaches may be preferable.^28^^,^^57^ However, our analysis highlights that these other methods may be limited as well, especially if they depend on a recorded positive SARS-CoV-2 test result since we found systematic differences between those with long COVID recorded, with and without a positive test result. The severity of the initial infection may also impact long COVID symptom presentation and potential recording in primary care.^58^

The level of long COVID captured in primary care coding is different to other studies, but the temporal trend we found with a decline in incidence over 2022 is consistent with work from the ONS and the USA.^12^^,^^59^ Vaccination and increased natural immunity is a likely contributing factor in this decline.^18^^,^^60^, ^61^, ^62^, ^63^, ^64^, ^65^, ^66^ However, there are methodological limitations in previous work as several studies are small^67^, ^68^, ^69^ or in self-selecting populations^70^, ^71^, ^72^, ^73^ and it is difficult to disentangle the relative contribution of vaccines, variants, and reinfections and how these affect the probability of long-term complications.^74^, ^75^, ^76^, ^77^

As the COVID-19 situation evolves in the UK, surveillance methods are changing and data collection for the ONS COVID-19 infection survey was paused in March 2023. This limits the possible data sources for monitoring and understanding long COVID. We have shown that long COVID clinical coding is limited in comparison to nationally representative random sampling such as the ONS-CIS, but EHRs have the potential to be an important resource for long COVID research. However, until we can better understand the reasons for the under-reporting of cases in primary care, this potential will not be realised.

One attractive solution in EHRs is to develop an alternative algorithm of detection method from rich data for identifying people with long COVID through identification of symptoms associated with long COVID.^28^^,^^29^^,^^78^ These methods often include a positive SARS-CoV-2 test result as a prerequisite, however our work shows that this would fail to detect 59% of the recorded long COVID coded individuals in our cohort. A combination of detection methods is therefore a necessity in future EHR long COVID research.

Data from multiple sources is needed to validate the definitions of long COVID between studies and establish a consistent definition so that research findings are generalisable outside of a specific study with a specific outcome definition. Validation of outcome measures is needed to better capture cases. Future research should combine routinely collected data with more granular detailed survey responses^12^^,^^36^^,^^77^ to better understand the differences between these data sources and triangulate evidence.

Despite the difficulties in researching vaccine effectiveness on long COVID, it is an important question to understand. It is unclear what role vaccination had in the protection against long COVID, beyond reduced risk of any infection. Further analysis could expand on research of heterogeneous vaccine mixing and different vaccine schedules and the impact these had on infections,^79^, ^80^, ^81^, ^82^ and whether these possible benefits confer to reduced long term COVID-19 incidence or symptom burden.

National survey data suggests that many people in the UK suffer with long COVID, but relatively few cases are recorded in primary care. We have shown that using EHR diagnostic or referral codes unfortunately has major limitations in identifying and ascertaining true cases and timing that severely limit its utility in shedding light on causal pathways to prevent or treat Long COVID.

## Contributors

Author contributions were as follows: Conceptualisation (ADH, LAT, MM, MJ, AB, L-YL, OC, EH, RME), data curation (ADH, BFCB–C, CB, JP, SCJB, ID, RME, BG, OSC), formal analysis (ADH), funding acquisition (BG, EH, RME), methodology (ADH, JT, LAT, MM, MJ, AB, L-YL, OC, WAD, REC, YW, AJW, WH, BFCB–C, CB, JP, SCJB, ID, RME, BG, AM, BM, OSC, EH, RME), project administration (BG, AM, BM, EH, RME), software (BFCB–C, CB, JP, SCJB, ID, BG, OSC), supervision (EH, RME), visualisation (ADH, EH, RME), writing – original draft (ADH). Access to the underlying identifiable and potentially re-identifiable pseudonymised electronic health record data is tightly governed by various legislative and regulatory frameworks, and restricted by best practice. The data in the NHS England OpenSAFELY COVID-19 service is drawn from General Practice data across England where TPP is the data processor. TPP developers initiate an automated process to create pseudonymised records in the core OpenSAFELY database, which are copies of key structured data tables in the identifiable records. These pseudonymised records are linked onto key external data resources that have also been pseudonymised via SHA-512 one-way hashing of NHS numbers using a shared salt. University of Oxford, Bennett Institute for Applied Data Science developers and PIs, who hold contracts with NHS England, have access to the OpenSAFELY pseudonymised data tables to develop the OpenSAFELY tools. These tools in turn enable researchers with OpenSAFELY data access agreements to write and execute code for data management and data analysis without direct access to the underlying raw pseudonymised patient data, and to review the outputs of this code. All code for the full data management pipeline — from raw data to completed results for this analysis — and for the OpenSAFELY platform as a whole is available for review at github. com/OpenSAFELY. For this study, ADH, REC and EH verified the underlying data. All authors (except WAD) were able to access the data through the OpenSAFELY platform as described above All authors reviewed and approved the final manuscript.

## Data sharing statement

All data management and analysis code is available on GitHub (https://github.com/opensafely/openprompt-vaccine-long-covid). Data management and analysis was performed using the OpenSAFELY software libraries and Python 3. All analysis was conducted in R version 4.2.1.^83^ All codelists used to define conditions and variables are openly available online at www.OpenCodelists.org for inspection and re-use. Definitions used in this study reuse codelists developed for published studies.^27^^,^^42^^,^^84^^,^^85^

Access to the underlying identifiable and potentially re-identifiable pseudonymised electronic health record data is tightly governed by various legislative and regulatory frameworks, and restricted by best practice. The data in the NHS England OpenSAFELY COVID-19 service is drawn from General Practice data across England where TPP is the data processor.

TPP developers initiate an automated process to create pseudonymised records in the core OpenSAFELY database, which are copies of key structured data tables in the identifiable records. These pseudonymised records are linked onto key external data resources that have also been pseudonymised via SHA-512 one-way hashing of NHS numbers using a shared salt. University of Oxford, Bennett Institute for Applied Data Science developers and PIs, who hold contracts with NHS England, have access to the OpenSAFELY pseudonymised data tables to develop the OpenSAFELY tools.

These tools in turn enable researchers with OpenSAFELY data access agreements to write and execute code for data management and data analysis without direct access to the underlying raw pseudonymised patient data, and to review the outputs of this code. All code for the full data management pipeline — from raw data to completed results for this analysis — and for the OpenSAFELY platform as a whole is available for review at github. com/OpenSAFELY.

## Declaration of interests

All authors have completed the ICMJE uniform disclosure form at www.icmje.org/coi_disclosure.pdf and declare the following: LAT reports grants from MRC, Wellcome, NIHR in the past 3 years. MJ reports funding from BMGF, Gavi, RCUK, BMGF, WHO, Gavi, Wellcome Trust, European Commission, InnoHK, TFGH, CDC to their institution over the past 3 years. AB has received consultancy fees within the past 3 years from AstraZeneca, Takeda, Daiichi-Sankyo, Eisai, Roche, Novartis, Idorsia & Rythmn. CB & JP are employees of TPP (Leeds) Ltd who provide SystmOne and process data for OpenSafely under the instruction of NHS England. REC holds personal shares in AstraZeneca unrelated to this work. BG has received research funding from the Bennett Foundation, the Laura and John Arnold Foundation, the NHS National Institute for Health Research (NIHR), the NIHR School of Primary Care Research, NHS England, the NIHR Oxford Biomedical Research Centre, the Mohn-Westlake Foundation, NIHR Applied Research Collaboration Oxford and Thames Valley, the Wellcome Trust, the Good Thinking Foundation, Health Data Research UK, the Health Foundation, the World Health Organisation, UKRI MRC, Asthma UK, the British Lung Foundation, and the Longitudinal Health and Wellbeing strand of the National Core Studies programme; he has previously been a Non-Executive Director at NHS Digital; he also receives personal income from speaking and writing for lay audiences on the misuse of science. AM has represented the RCGP in the health informatics group and the Profession Advisory Group that advises on access to GP Data for Pandemic Planning and Research (GDPPR); the latter was a paid role. AM is a former employee and interim Chief Medical Officer of NHS Digital. AM has consulted for health care vendors, the last time in 2022; the companies consulted in the last 3 years have no relationship to OpenSAFELY. BMK is also employed by NHS England working on medicines policy and clinical lead for primary care medicines data. All other authors declare no competing interests.

## Appendix A Supplementary data

Supplementary methods, text, Figs. S1–S10, and Tables S1–S3

OS-collaborative list
