# Supplementary material for: Clinical coding of long COVID in primary care 2020–2023 in a cohort of 19 million adults: an OpenSAFELY analysis
Source: eClinicalMedicine. 2024 May 17;72:102638. doi: 10.1016/j.eclinm.2024.102638 (PMC11127160; doi:10.1016/j.eclinm.2024.102638)
Supplement: Supplementary methods, text, Figs. S1–S10, and Tables S1–S3 [file mmc1.pdf]

Appendix – Clinical coding of long COVID in primary care 2020-2023 in a cohort of 19 million adults: an OpenSAFELY analysis

Supplementary methods **2**
Supplementary text **5**
Supplementary figures **7**
Supplementary tables **16**

Supplementary methods

Information governance and ethical approval

NHS England is the data controller of the NHS England OpenSAFELY COVID-19 Service. TPP is the data processor; all study authors using OpenSAFELY have the approval of NHS England. This implementation of OpenSAFELY is hosted within the TPP environment which is accredited to the ISO 27001 information security standard and is NHS IG Toolkit compliant. Patient data has been pseudonymised for analysis and linkage using industry standard cryptographic hashing techniques; all pseudonymised datasets transmitted for linkage onto OpenSAFELY are encrypted; access to the NHS England OpenSAFELY COVID-19 service is via a virtual private network (VPN) connection; the researchers hold contracts with NHS England and only access the platform to initiate database queries and statistical models; all database activity is logged; only aggregate statistical outputs leave the platform environment following best practice for anonymisation of results such as statistical disclosure control for low cell counts.

The service adheres to the obligations of the UK General Data Protection Regulation (UK GDPR) and the Data Protection Act 2018. The service previously operated under notices initially issued in February 2020 by the the Secretary of State under Regulation 3(4) of the Health Service (Control of Patient Information) Regulations 2002 (COPI Regulations), which required organisations to process confidential patient information for COVID-19 purposes; this set aside the requirement for patient consent. As of 1 July 2023, the Secretary of State has requested that NHS England continue to operate the Service under the COVID-19 Directions 2020. In some cases of data sharing, the common law duty of confidence is met using, for example, patient consent or support from the Health Research Authority Confidentiality Advisory Group.

Taken together, these provide the legal bases to link patient datasets using the service. GP practices, which provide access to the primary care data, are required to share relevant health information to support the public health response to the pandemic, and have been informed of how the service operates.

This research is part of the OpenPROMPT study “Quality-of-life in patients with long COVID: harnessing the scale of big data to quantify the health and economic costs” which has ethical approval from HRA and Health and Care Research Wales (HCRW) (IRAS project ID 304354). The Study Coordination Centre has obtained approval from the LSHTM Research Ethics Committee (ref 28030), as well as a favourable opinion from the South Central—Berkshire B Research Ethics Committee (ref 22/SC/0198).

Hierarchical long COVID definition

We separated SNOMED-CT codes that indicated long COVID in a patient health record into those that were clinical diagnoses, and other referral/assessment codes. We established a hierarchical search for the first record of long COVID because we determined that a diagnosis code gave stronger evidence of the presence of long COVID, and therefore a more accurate timing of the record as well. We therefore initially searched a patient’s record for diagnosis code first, and if none existed then searched for a referral/assessment code. If neither code type existed then that participant was classified as not having long COVID.

Secondary cohort
In a separate cohort, we investigated the impact of having EHR-recorded long COVID on further vaccination rates. We therefore developed a secondary cohort that includes only those with a record of long COVID and follows up until January 2023 or loss to follow-up, and we summarised vaccine coverage in this cohort as of January 2023.

The secondary cohort included all patients with a record of Long COVID before they had a vaccination. Individuals entered this cohort at the time of a diagnosis (or referral) for long COVID. Individuals were excluded if they have already received a vaccination at this point. We included all individuals who met this criteria from 1st November 2020 to 22nd October 2021, when the first booster vaccination was delivered (1). We followed up this cohort until January 2023 and measured the number of vaccine doses these individuals received over the study period. We calculated the rate of vaccination in this cohort and compared this to the overall rate of vaccination in the primary cohort.

Long COVID after hospitalisation
We also defined Long COVID outcomes dependent on previous SARS-COV-2 history. All records of Long COVID were further divided into the following groups:

1. Long COVID record in primary care following hospitalisation with COVID-19
2. Long COVID record in primary care following a positive SARS-COV-2 test (SGSS)
3. Long COVID record in primary care with no further information

Definition of hospitalisation with COVID-19: Data on Hospital Episode Statistics are available including the ICD-10 codes recorded during non-emergency hospital admission. Hospitalisation with COVID-19 was identified from Hospital Episode Statistics Admitted Patient Care (HES-APC) data available in OpenSAFELY-TPP. The following ICD-10 codes were used to define COVID-19 hospitalisations (at any point in the hospitalisation record, i.e., not primary diagnosis only):

icd10_code	
U071	covid19 virus identified
U072	covid19 virus not identified

Although ICD-10 code U072 refers to SARS-COV-2 *not* being identified, this code was used when COVID-19 is suspected clinically or epidemiologically but the laboratory test is inconclusive or negative. All previous SARS-COV-2 test results and/or hospitalisation records at least 12 weeks prior to the Long COVID record were considered when classifying Long COVID outcomes.

Covariates
All codelists used to define covariates are available in the GitHub repository for this project https://github.com/opensafely/openprompt-vaccine-long-covid

**Stratifier**	**Definition**
Number of vaccination doses	Time updated stratification variable where individuals move from 0 to 6 doses as they become vaccinated (as defined above)

Stratifier	Definition
First vaccine dose	Vaccines distributed in the UK were mostly either BNT162b2 or ChAdOx1 administered as part of the national covid-19 vaccine roll-out. We will compare long COVID rates by the manufacturer of the first vaccine dose received by individuals.
Receipt of mRNA vaccine	Vaccines were classified as either mRNA (Pfizer. Comirnarty, Moderna, Spikevax) or not. It is possible that these different vaccine types offered different protection against COVID-19 and related outcomes.<br><br>We will create a time-updated variable indicating when someone received an mRNA vaccine with three levels: No vaccine, non-mRNA only, mRNA received
Age	Age at study entry
Sex	Male or female, excluding individuals with missing data
Region	Geographical area as categorised by 9 English regions categorised as North East, North West, Yorkshire and the Humber, East Midlands, West Midlands, East, London, South East, South West
Ethnicity	Five categories, obtained from 16 (White, Black, South Asian, Mixed, Other).<br>Assessed from primary care records and supplemented with HES APC records (2)
Deprivation	Index of multiple deprivation (IMD) quintile based on lower super output area
History of post-viral fatigue	Binary variable.<br>Codelist is available (3) from previous Long COVID research
High risk category for developing complication from COVID-19	Binary variable.<br>Codelist is available (4) from previous vaccine research (5)
Level of multimorbidity	Categorised as 0, 1, 2+.<br><br>Comorbidities are assessed at study entry. Relevant comorbidities will be defined based on previous research of risk factors for Long COVID in OpenSAFELY (6). A previous code 6 months to 5 years before March 2020 for one or more of: diabetes; cancer; haematological cancer; asthma; chronic respiratory disease; chronic cardiac disease; chronic liver disease; stroke or dementia; other neurological condition; organ transplant; dysplasia; rheumatoid arthritis, systemic lupus erythematosus or psoriasis; or other immunosuppressive conditions. Those with no relevant code for a condition will be assumed not to have that condition. Number of conditions were categorised into “0”, “1”, and “2 or more”..

Statistical analysis

The crude rate $\left(\frac{d}{T}\right)$ was expressed per 100,000 person-years where d is the number of events and T the total follow-up time of observation, with 95% confidence intervals from $rate/EF$ to $rate \times EF$ where $EF = exp\left(1.96/\sqrt{d}\right)$ (7).

We visualised the temporal dynamics of long COVID captured in EHRs on a weekly scale and further summarised the number of recorded cases on a daily scale. In plots, we stratified the weekly totals by diagnosis or referral codes, the three most prevalent long COVID SNOMED codes in the data, vaccination status and sex. We compared the dynamics of long COVID to the total recorded cases from the UK Coronavirus dashboard (8).

Supplementary Methods References

1. Vaccinations in the UK | Coronavirus in the UK [Internet]. 2023 [cited 2023 Jan 27]. Available from: https://coronavirus.data.gov.uk/details/vaccinations
2. OpenCodelists: ethnicity (SNOMED) [Internet]. [cited 2023 Feb 15]. Available from: https://www.opencodelists.org/codelist/opensafely/ethnicity-snomed-0removed/2e641f61/
3. Clinical coding of long COVID in English primary care: a federated analysis of 58 million patient records in situ using OpenSAFELY [Internet]. OpenSAFELY; 2022 [cited 2023 Feb 15]. Available from: https://github.com/opensafely/long-covid/blob/d44629d020e2687171264d6b42bb81a6aa92f07e/codelists/user-alex-walker-post-viral-syndrome.csv
4. OpenCodelists: High Risk from COVID-19 code [Internet]. [cited 2023 Feb 15]. Available from: https://www.opencodelists.org/codelist/primis-covid19-vacc-uptake/shield/v1/#full-list
5. Curtis HJ, Inglesby P, MacKenna B, Croker R, Hulme WJ, Rentsch CT, et al. Recording of 'COVID-19 vaccine declined': a cohort study on 57.9 million National Health Service patients' records in situ using OpenSAFELY, England, 8 December 2020 to 25 May 2021. Eurosurveillance. 2022 Aug 18;27(33):2100885.
6. Thompson EJ, Williams DM, Walker AJ, Mitchell RE, Niedzwiedz CL, Yang TC, et al. Long COVID burden and risk factors in 10 UK longitudinal studies and electronic health records. Nat Commun. 2022 Jun 28;13(1):3528.
7. Kirkwood BR, Sterne JAC, Kirkwood BR. Essential medical statistics. 2nd ed. Malden, Mass: Blackwell Science; 2003. 501 p.
8. Cases in England | Coronavirus in the UK [Internet]. 2023 [cited 2023 Apr 27]. Available from: https://coronavirus.data.gov.uk/details/cases?areaType=nation&areaName=England

Supplementary text

Recording long COVID in vaccinated groups
We compared the rate of recorded long COVID between people with 0, 1, 2, 3+ SARS-COV-2 vaccinations. Due to the uncertainty in the accuracy and timing of long COVID onset in our descriptive analysis, these results are not generalisable and do not reflect any meaningful causal relationship between SARS-COV-2 vaccinations and the probability of new onset long COVID.

Recorded long COVID is an imprecise measure of the actual incidence of the condition. These results are therefore presented as simple comparisons of an imperfect outcome measure and no causal or aetiological conclusions can be drawn from them, however we believe they still have value to inform future research and they have been presented for completeness.

In the primary cohort, followed up until they received a long COVID code, the crude rate of recorded long COVID was lowest for people with 3 or more vaccine doses (103.5 per 100,000 person-years; 95% CI: 101.5-105) (**Figure S7, Table S1**). We estimated the rates in different vaccine dose groups adjusted for age, sex, region and variant, but this is not equivalent to a vaccine effect estimate. The rate of recorded long COVID was 0.85 (95% CI: 0.73-0.99) times lower at least 14 weeks after one vaccine dose, 0.58 (95% CI: 0.5-0.68) times lower after 2 doses, and 0.15 (95% CI: 0.12-0.18) times lower after 3 or more doses, with similar patterns for long COVID diagnosis codes only (**Figure S7, Table S2**). The rate of recording of long COVID was lower in people who received an mRNA-based vaccine for their first dose (RR 0.41; 95% CI: 0.3-0.47) compared to unvaccinated. Those who received adenovirus-based (or other non mRNA formulated vaccines) as a first dose still had lower rates of long COVID in the analysis but with a rate ratio closer to null (0.87; 95% CI: 0.77-0.99). To quality assure the analysis we repeated the models with COVID-19 hospitalisation as an outcome and found associations with age, sex, and vaccination that are consistent with previous research (**Figure S7**).

There is a lag between vaccination and protection from infection, and a further lag until a diagnosis or referral for long COVID can be made. In our main analysis we assumed that this gap is 14 weeks in total. In a sensitivity analysis we expanded this time gap to 18 or 26 weeks. The findings from these results were consistent with our main findings of reduced rates of long COVID with increasing vaccine doses, and a lower rate ratio for those receiving mRNA than non-mRNA vaccines as a first dose (**Figure S8**). We also repeated the analysis stratified by three broad categorisations of the dominant circulating variant of SARS-COV-2 (wild/alpha, delta, omicron). We found that the rate ratios for the effect of vaccination were lowest for long COVID recording during the wild/alpha and delta period and higher during omicron, but were consistently lowest in those with 3+ vaccine doses (**Figure S9**).

Secondary cohort analysis
In a secondary cohort we continued to follow people after their long COVID record. In this exploratory analysis across we found that the percentage of unvaccinated people was greater in those with a previous long COVID record compared to those without, and this difference was largest in the youngest (18-29) and oldest (70+) age groups (**Figure S10**).

Supplementary figures

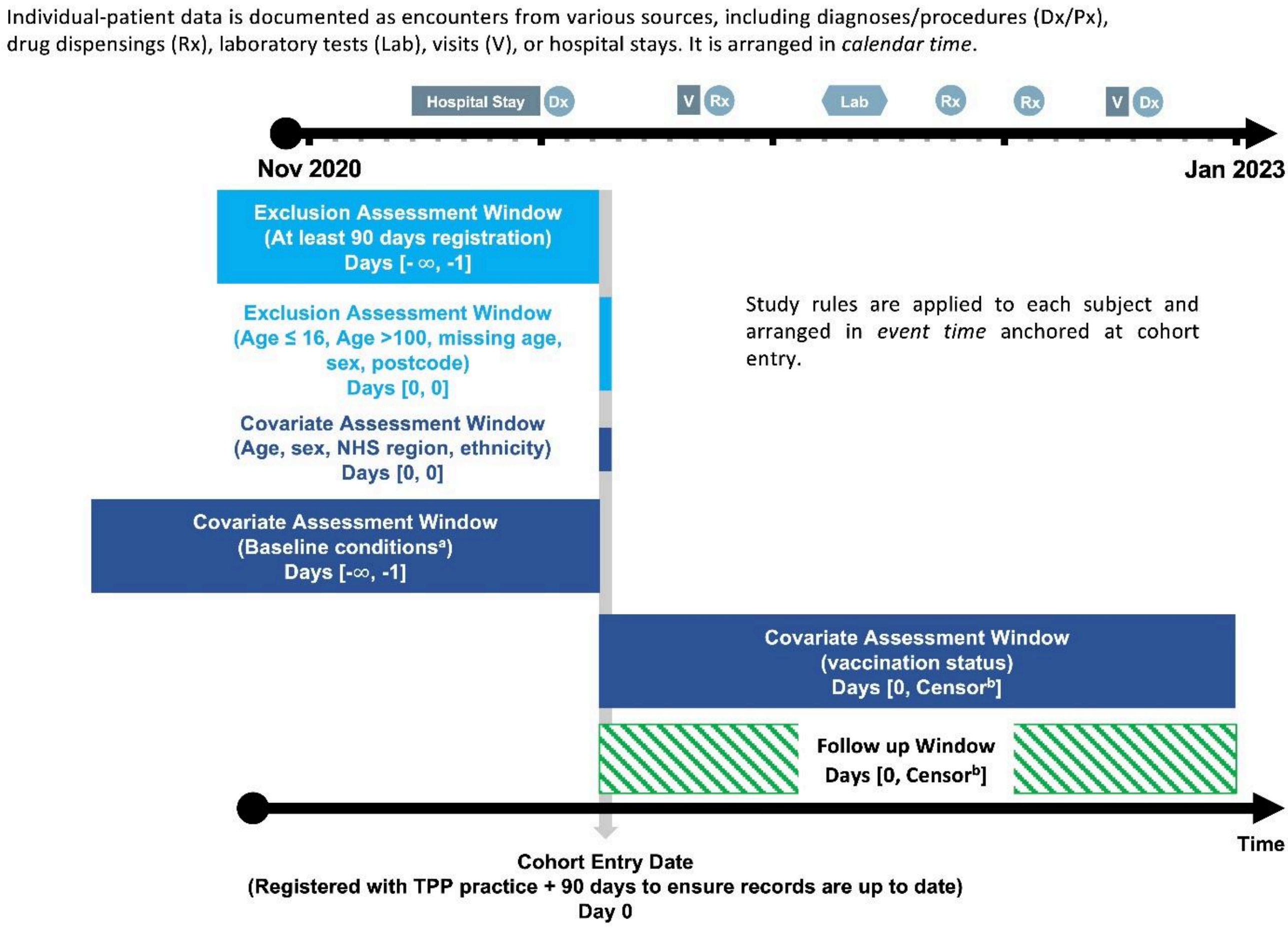

a. Baseline conditions included: high/moderate risk category for developing complication from COVID-19, number of comorbidities
b. Earliest of: outcome of interest (recorded long COVID), end of registration, death, end of the study period

Figure S1: Study design diagram for the primary cohort

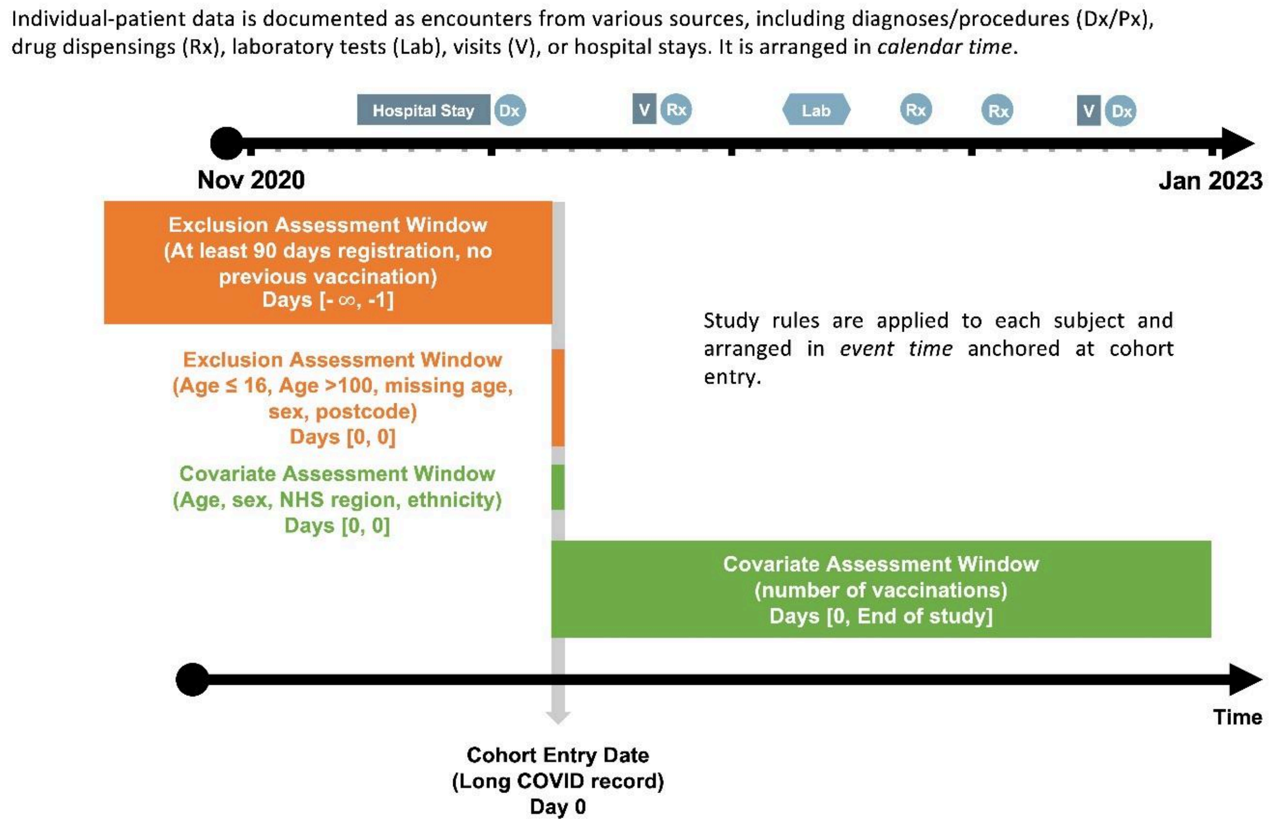

Figure S2: Study design diagram for the secondary analysis of vaccination *after* EHR recorded long COVID

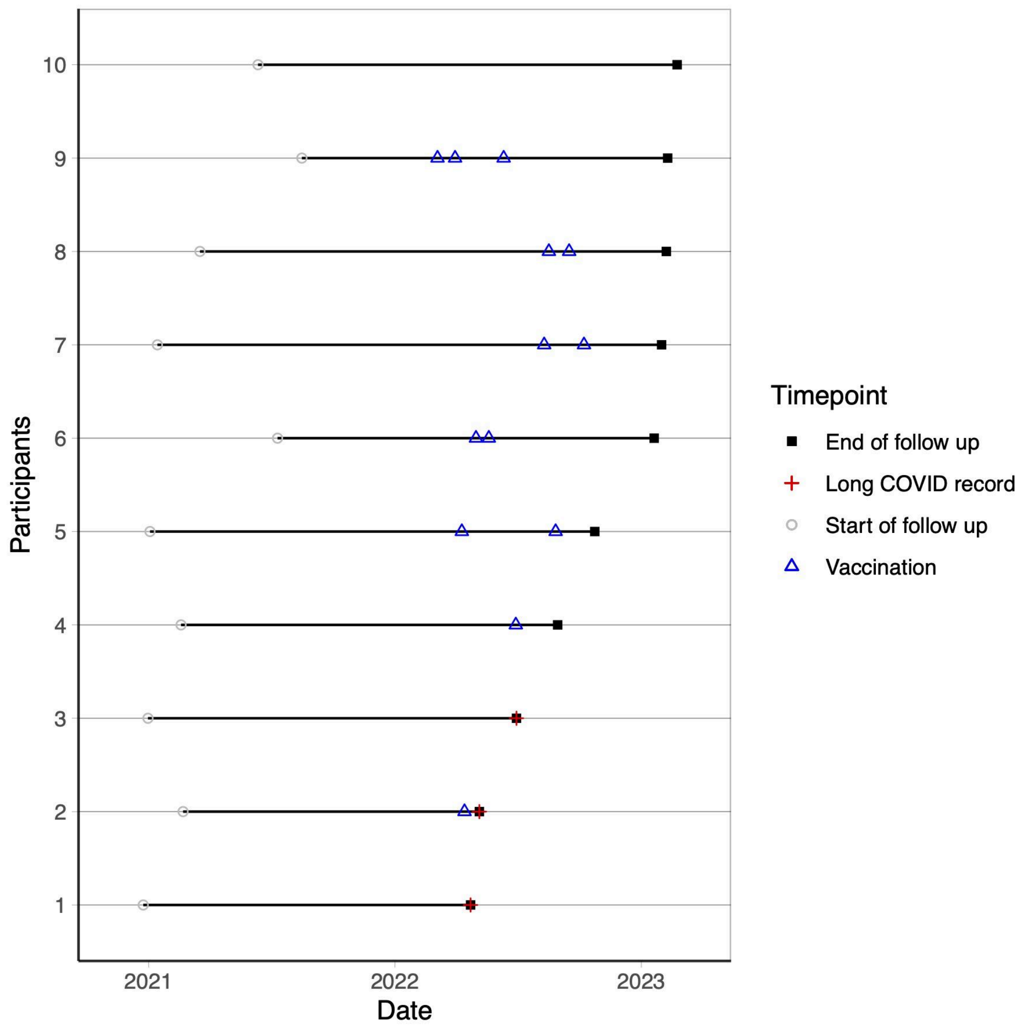

Figure S3: Schematic of the study design and recorded data for 10 hypothetical study participants

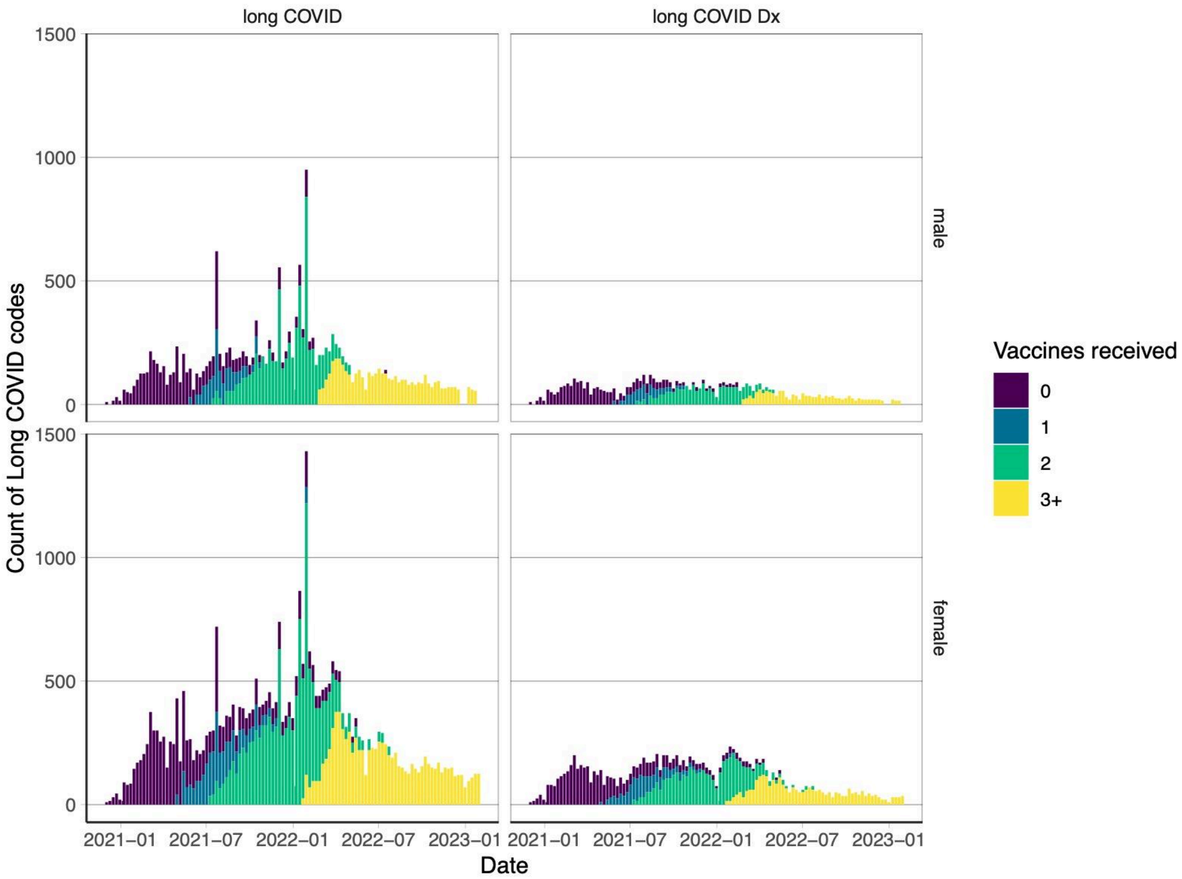

Figure S4: Bar chart of the weekly number of incident (new) long COVID records between November 2020 and January 2023, stratified by vaccination status, sex and code type. Left hand panels include any long COVID code in the codelist. Right hand panels ("long COVID Dx") includes codes relating to diagnosis of long COVID only.

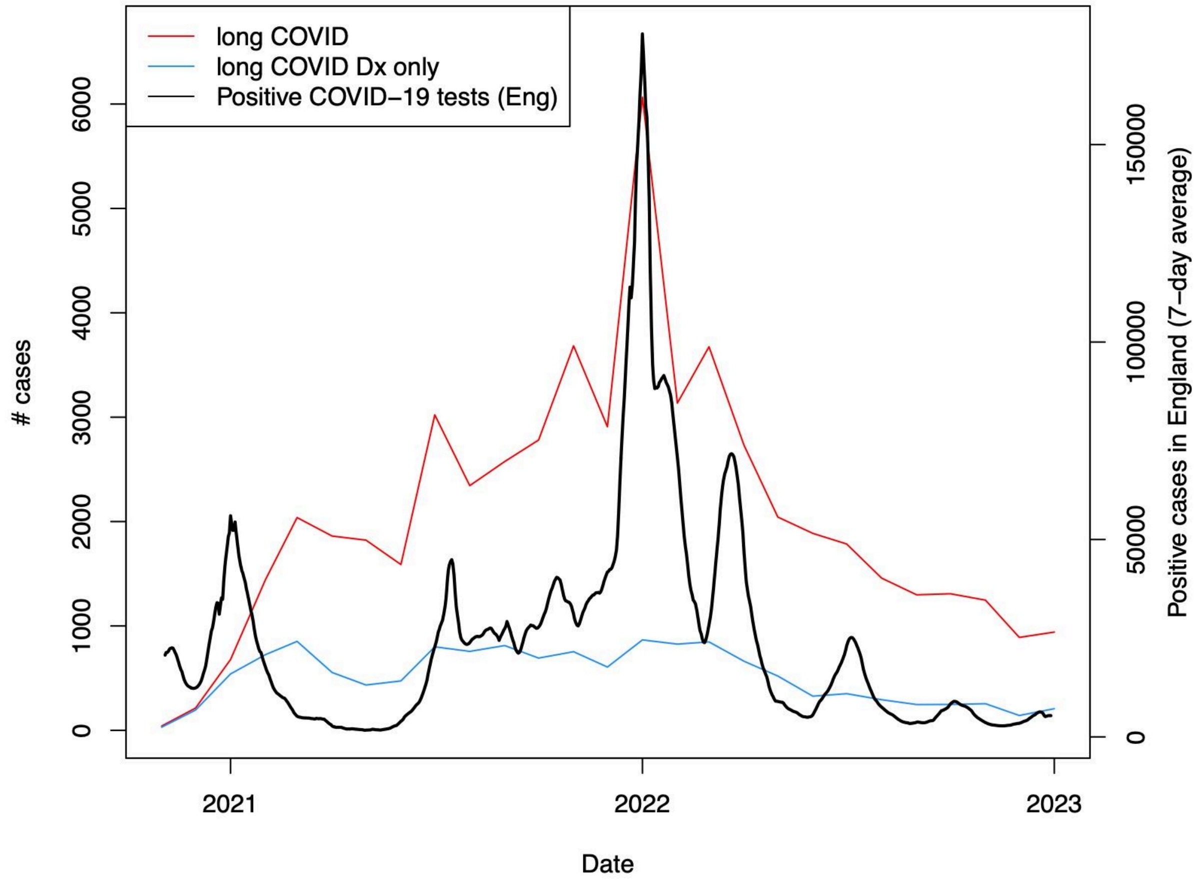

Figure S5: Monthly count of new long COVID records over time for any long COVID code (red), or long COVID diagnosis codes only (blue) (left-hand axis). These trends are plotted alongside the 7-day rolling average of positive COVID-19 tests available from the UK Health Security Agency (right-hand axis). To define long COVID, records were searched for a diagnosis code first (Any long COVID diagnosis code), if no code existed then we searched for a referral code (Any long COVID code). If neither code existed then the individual was classified as not having long COVID.

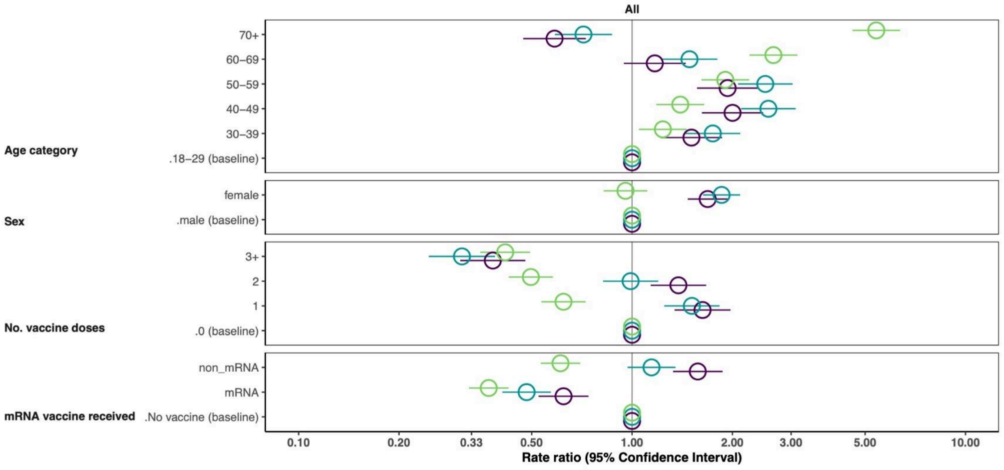

		All<br>All Long COVID		All<br>Long COVID diagnoses		All<br>COVID−19 hospitalisation	
		RR	95% CI	RR	95% CI	RR	95% CI
Age category	70+	0.59	(0.47–0.73)	0.71	(0.59–0.87)	5.4	(4.6–6.4)
	60–69	1.2	(0.94–1.4)	1.5	(1.2–1.8)	2.7	(2.2–3.1)
	50–59	1.9	(1.6–2.4)	2.5	(2.1–3)	1.9	(1.6–2.2)
	40–49	2	(1.6–2.5)	2.6	(2.1–3.1)	1.4	(1.2–1.6)
	30–39	1.5	(1.2–1.9)	1.7	(1.4–2.1)	1.2	(1–1.5)
	.18–29 (baseline)	1	(1–1)	1	(1–1)	1	(1–1)
Sex	female	1.7	(1.5–1.9)	1.9	(1.6–2.1)	0.96	(0.82–1.1)
	.male (baseline)	1	(1–1)	1	(1–1)	1	(1–1)
No. vaccine doses	3+	0.38	(0.31–0.48)	0.31	(0.25–0.39)	0.42	(0.35–0.49)
	2	1.4	(1.1–1.7)	0.99	(0.82–1.2)	0.5	(0.43–0.58)
	1	1.6	(1.3–2)	1.5	(1.2–1.8)	0.62	(0.53–0.73)
	.0 (baseline)	1	(1–1)	1	(1–1)	1	(1–1)
mRNA vaccine received	non_mRNA	1.6	(1.3–1.9)	1.1	(0.97–1.3)	0.61	(0.53–0.7)
	mRNA	0.62	(0.52–0.74)	0.48	(0.41–0.57)	0.37	(0.32–0.43)
	.No vaccine (baseline)	1	(1–1)	1	(1–1)	1	(1–1)

Figure S6: Crude rate ratios for records of long COVID and COVID-19 hospitalisation. Rate ratios are estimated from negative binomial regression models with a single covariate in each model (age category, sex, number of vaccine doses or whether the first does was mRNA). To define long COVID, records were searched for a diagnosis code first (Any long COVID diagnosis code), if no code existed then we searched for a referral code (Any long COVID code). If neither code existed then the individual was classified as not having long COVID.

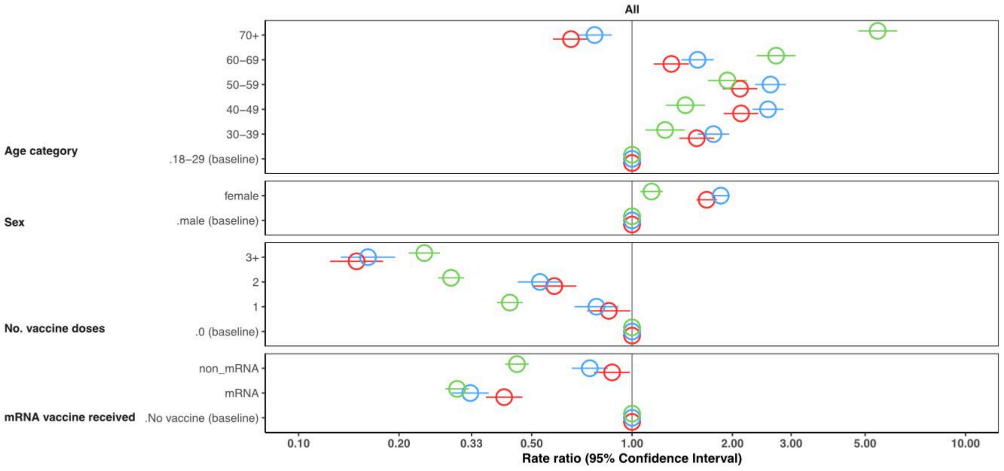

		All<br>All Long COVID<br>RR	 <br>95% CI	All<br>Long COVID diagnoses<br>RR	 <br>95% CI	All<br>COVID−19 hospitalisation<br>RR	 <br>95% CI
	70+	0.66	(0.58–0.74)	0.77	(0.69–0.87)	5.5	(4.8–6.2)
	60–69	1.3	(1.2–1.5)	1.6	(1.4–1.8)	2.7	(2.4–3.1)
	50–59	2.1	(1.9–2.4)	2.6	(2.3–2.9)	1.9	(1.7–2.2)
	40–49	2.1	(1.9–2.4)	2.6	(2.3–2.8)	1.4	(1.3–1.7)
	30–39	1.6	(1.4–1.8)	1.8	(1.6–2)	1.3	(1.1–1.4)
**Age category**	.18–29 (baseline)	1	(1–1)	1	(1–1)	1	(1–1)
	female	1.7	(1.6–1.8)	1.8	(1.7–2)	1.1	(1.1–1.2)
**Sex**	.male (baseline)	1	(1–1)	1	(1–1)	1	(1–1)
	3+	0.15	(0.12–0.18)	0.16	(0.13–0.19)	0.24	(0.21–0.27)
	2	0.58	(0.5–0.68)	0.53	(0.45–0.62)	0.29	(0.26–0.31)
	1	0.85	(0.73–0.99)	0.78	(0.67–0.91)	0.43	(0.39–0.47)
**No. vaccine doses**	.0 (baseline)	1	(1–1)	1	(1–1)	1	(1–1)
	non_mRNA	0.87	(0.77–0.99)	0.75	(0.66–0.85)	0.45	(0.42–0.49)
	mRNA	0.41	(0.36–0.47)	0.33	(0.29–0.37)	0.3	(0.28–0.32)
**mRNA vaccine received**	.No vaccine (baseline)	1	(1–1)	1	(1–1)	1	(1–1)

Outcome
- All Long COVID
- Long COVID diagnoses
- COVID−19 hospitalisation

Figure S7: Adjusted rate ratios for records of long COVID and COVID-19 hospitalisation. Rate ratios are estimated from negative binomial regression models adjusted for age, sex, 9 NHS regions of England, and the dominant variant circulating. To define long COVID, records were searched for a diagnosis code first (Any long COVID diagnosis code), if no code existed then we searched for a referral code (Any long COVID code). If neither code existed then the individual was classified as not having long COVID.

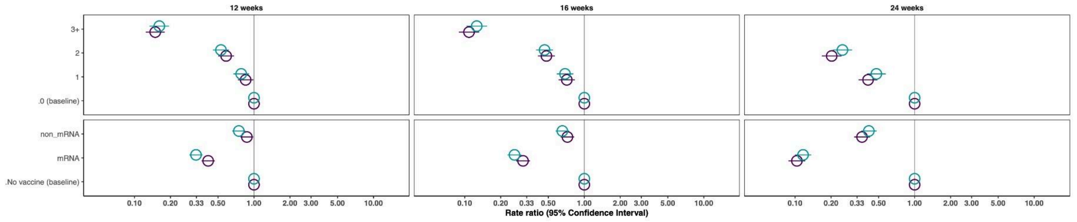

		12 weeks All Long COVID RR	12 weeks All Long COVID 95% CI	12 weeks Long COVID diagnoses RR	12 weeks Long COVID diagnoses 95% CI	16 weeks All Long COVID RR	16 weeks All Long COVID 95% CI	16 weeks Long COVID diagnoses RR	16 weeks Long COVID diagnoses 95% CI	24 weeks All Long COVID RR	24 weeks All Long COVID 95% CI	24 weeks Long COVID diagnoses RR	24 weeks Long COVID diagnoses 95% CI
	3+	0.15	(0.12–0.18)	0.16	(0.13–0.19)	0.11	(0.09–0.13)	0.13	(0.1–0.15)	0.03	(0.02–0.04)	0.04	(0.03–0.05)
	2	0.58	(0.5–0.68)	0.53	(0.45–0.62)	0.48	(0.41–0.57)	0.47	(0.4–0.55)	0.2	(0.17–0.25)	0.25	(0.21–0.3)
	1	0.85	(0.73–0.99)	0.78	(0.67–0.91)	0.71	(0.61–0.84)	0.69	(0.59–0.81)	0.41	(0.34–0.49)	0.48	(0.4–0.57)
No. vaccine doses	.0 (baseline)	1	(1–1)	1	(1–1)	1	(1–1)	1	(1–1)	1	(1–1)	1	(1–1)
	non_mRNA	0.87	(0.77–0.99)	0.75	(0.66–0.85)	0.72	(0.63–0.82)	0.66	(0.58–0.75)	0.36	(0.31–0.42)	0.41	(0.36–0.48)
	mRNA	0.41	(0.36–0.47)	0.33	(0.29–0.37)	0.31	(0.27–0.35)	0.26	(0.23–0.3)	0.1	(0.09–0.12)	0.12	(0.1–0.14)
mRNA vaccine received	.No vaccine (baseline)	1	(1–1)	1	(1–1)	1	(1–1)	1	(1–1)	1	(1–1)	1	(1–1)

Figure S8: Sensitivity analysis of negative binomial models for vaccine covariates. Results show the rate ratios for records of long COVID and COVID-19 hospitalisation. Rate ratios are estimated from negative binomial regression models adjusted for age, sex, 9 NHS regions of England, and the dominant variant circulating. The models are run under three different data management scenarios. The first ("12 weeks") is the primary analysis, the others show results when the gap between vaccine date and long COVID/end of follow up is extended to 16 and 24 weeks. To define long COVID, records were searched for a diagnosis code first (Any long COVID diagnosis code), if no code existed then we searched for a referral code (Any long COVID code). If neither code existed then the individual was classified as not having long COVID.

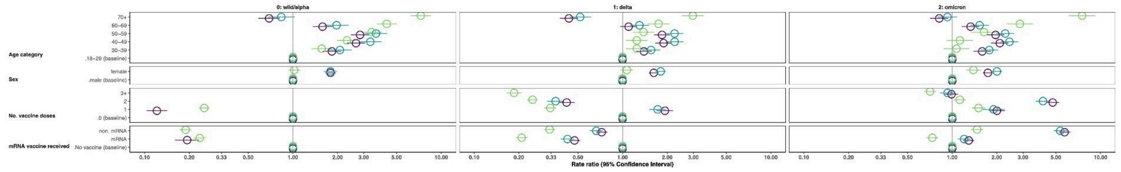

		0: wild/alpha All Long COVID RR	95% CI	0: wild/alpha Long COVID diagnoses RR	95% CI	0: wild/alpha COVID−19 hospitalisation RR	95% CI	1: delta All Long COVID RR	95% CI	1: delta Long COVID diagnoses RR	95% CI	1: delta COVID−19 hospitalisation RR	95% CI
	70+	0.69	(0.59–0.81)	0.83	(0.68–1)	7.3	(6.2–8.5)	0.43	(0.38–0.5)	0.52	(0.44–0.61)	3	(2.5–3.5)
	60–69	1.6	(1.4–1.8)	2	(1.6–2.4)	4.3	(3.7–5)	1.1	(0.96–1.3)	1.3	(1.1–1.5)	1.7	(1.5–2.1)
	50–59	2.8	(2.5–3.3)	3.6	(3–4.3)	3.4	(2.9–4)	1.8	(1.6–2.1)	2.2	(2–2.6)	1.4	(1.2–1.6)
	40–49	2.7	(2.3–3.1)	3.3	(2.8–4)	2.3	(2–2.7)	1.9	(1.7–2.1)	2.2	(2–2.6)	1.2	(1.1–1.5)
	30–39	1.8	(1.6–2.1)	2.1	(1.7–2.5)	1.6	(1.3–1.8)	1.4	(1.2–1.6)	1.6	(1.4–1.8)	1.2	(1.1–1.5)
Age category	.18–29 (baseline)	1	(1–1)	1	(1–1)	1	(1–1)	1	(1–1)	1	(1–1)	1	(1–1)
	female	1.8	(1.6–1.9)	1.8	(1.6–2)	1	(0.93–1.1)	1.6	(1.5–1.8)	1.8	(1.7–2)	1.1	(0.97–1.2)
Sex	.male (baseline)	1	(1–1)	1	(1–1)	1	(1–1)	1	(1–1)	1	(1–1)	1	(1–1)
	3+											0.19	(0.16–0.21)
	2					0.04	(0.04–0.05)	0.42	(0.37–0.48)	0.35	(0.31–0.4)	0.25	(0.23–0.27)
	1	0.12	(0.1–0.14)	0.05	(0.04–0.06)	0.25	(0.23–0.27)	1.9	(1.7–2.2)	1.7	(1.5–2)	0.33	(0.3–0.36)
No. vaccine doses	.0 (baseline)	1	(1–1)	1	(1–1)	1	(1–1)	1	(1–1)	1	(1–1)	1	(1–1)
	non_mRNA	0.04	(0.03–0.05)			0.19	(0.17–0.21)	0.72	(0.66–0.79)	0.66	(0.6–0.74)	0.32	(0.3–0.35)
	mRNA	0.19	(0.16–0.23)			0.24	(0.21–0.26)	0.47	(0.43–0.52)	0.43	(0.38–0.47)	0.21	(0.19–0.23)
mRNA vaccine received	.No vaccine (baseline)	1	(1–1)	1	(1–1)	1	(1–1)	1	(1–1)	1	(1–1)	1	(1–1)

		2: omicron All Long COVID RR	95% CI	2: omicron Long COVID diagnoses RR	95% CI	2: omicron COVID−19 hospitalisation RR	95% CI
	70+	0.82	(0.71–0.94)	0.93	(0.8–1.1)	7.5	(6.1–9.2)
	60–69	1.3	(1.2–1.5)	1.5	(1.3–1.8)	2.9	(2.3–3.5)
	50–59	2	(1.7–2.2)	2.3	(2–2.6)	1.6	(1.3–2)
	40–49	2.1	(1.8–2.4)	2.4	(2.1–2.8)	1.1	(0.92–1.4)
	30–39	1.6	(1.4–1.8)	1.8	(1.5–2)	1.1	(0.87–1.3)
Age category	.18–29 (baseline)	1	(1–1)	1	(1–1)	1	(1–1)
	female	1.7	(1.6–1.9)	2	(1.8–2.2)	1.4	(1.2–1.6)
Sex	.male (baseline)	1	(1–1)	1	(1–1)	1	(1–1)
	3+	0.99	(0.89–1.1)	0.93	(0.83–1)	0.71	(0.64–0.78)
	2	4.8	(4.3–5.3)	4.1	(3.6–4.6)	1.1	(1–1.2)
	1	2	(1.8–2.3)	1.9	(1.6–2.2)	1.5	(1.4–1.7)
No. vaccine doses	.0 (baseline)	1	(1–1)	1	(1–1)	1	(1–1)
	non_mRNA	5.7	(5.2–6.3)	5.3	(4.8–5.9)	1.5	(1.3–1.6)
	mRNA	1.3	(1.2–1.4)	1.2	(1.1–1.3)	0.73	(0.67–0.8)
mRNA vaccine received	.No vaccine (baseline)	1	(1–1)	1	(1–1)	1	(1–1)

Figure S9: Adjusted rate ratios for records of long COVID and COVID-19 hospitalisation stratified by the dominant variant circulating. Rate ratios are estimated from negative binomial regression models adjusted for age, sex, 9 NHS regions of England over three different time periods (wildtype/alpha, 1 November 2020 - 16 May 2021; Delta, 16 May 2021 - 1 December 2021; Omicron, 1 December 2021 - 31 Jan 2023). To define long COVID, records were searched for a diagnosis code first (Any long COVID diagnosis code), if no code existed then we searched for a referral code (Any long COVID code). If neither code existed then the individual was classified as not having long COVID.

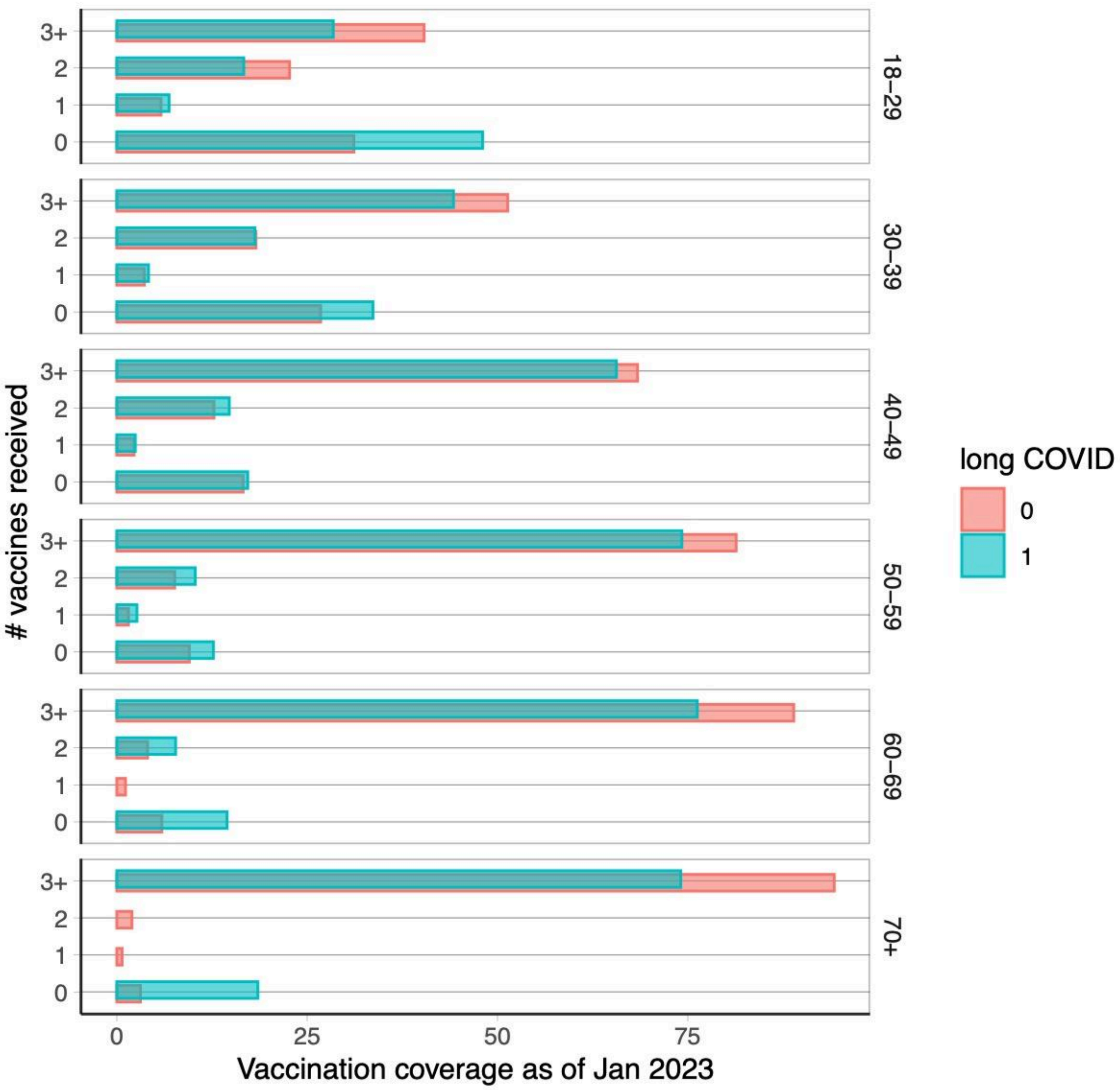

Figure S10: Coverage of vaccination *after* a record of long COVID (blue) compared to those that did not record long COVID (red). Bars show the percentage of people with 0, 1, 2, or 3+ vaccine doses as of January 2023

Supplementary tables

Table S1: Crude rates of incident long COVID codes in primary care between November 2020 and January 2023, stratified by demographic and clinical characteristics. All counts are rounded to the nearest 5 and counts less than 10 are redacted.

				Long COVID		Long COVID (diagnoses)	
Variable	Level	*N*	Follow-up	n	Rate per 100,000 person-years (95% CI)	n	Rate per 100,000 person-years (95% CI)
Sex	male	47,456,145	199.5	20,090	100.5 (99.5-102)	6,840	34.5 (33.5-35)
	female	49,229,120	199.5	35,375	177.5 (175.5-179)	13,185	66 (65-67)
Age category	18-29	17,027,985	77.5	7,585	98 (95.5-100)	2,335	30 (29-31.5)
	30-39	15,780,160	70	10,645	151.5 (148.5-154.5)	3,745	53.5 (51.5-55)
	40-49	15,863,125	66	13,595	205.5 (202-209)	5,125	77.5 (75.5-79.5)
	50-59	17,561,045	70	13,570	194 (191-197.5)	5,130	73.5 (71.5-75.5)
	60-69	13,929,120	54	6,350	118 (115-121)	2,355	43.5 (42-45.5)
	70+	16,523,830	61.5	3,715	60.5 (58.5-62.5)	1,340	22 (20.5-23)
Region	London	6,414,990	29	2,640	90.5 (87-94)	945	32.5 (30.5-34.5)
	East Midlands	16,670,975	68.5	7,815	114 (111.5-116.5)	3,280	48 (46-49.5)
	East	22,102,020	91	10,715	118 (115.5-120)	4,325	47.5 (46-49)
	North East	4,600,365	19	4,285	227 (220.5-234)	820	43.5 (40.5-46.5)
	North West	8,774,700	35.5	6,315	177.5 (173.5-182)	1,990	56 (53.5-58.5)
	South East	6,367,175	26	3,850	149 (144.5-154)	1,215	47 (44.5-49.5)
	South West	14,350,305	57.5	8,660	151 (147.5-154)	3,195	55.5 (54-57.5)
	West Midlands	3,667,965	15.5	1,765	112.5 (107.5-118)	740	47 (44-50.5)
	Yorkshire and The Humber	13,677,480	56.5	9,400	165.5 (162.5-169)	3,500	61.5 (59.5-64)
	NA	59,290	0.5	15	64.5 (40-104)	NA	-- --
Dominant SARS-COV-2 variant	0: wild/alpha	36,436,435	95	7,315	77 (75.5-79)	3,940	41.5 (40-43)
	1: delta	35,643,445	101.5	17,280	170 (167.5-173)	7,065	69.5 (68-71)
	2: omicron	24,605,385	202.5	30,870	152.5 (150.5-154)	9,020	44.5 (43.5-45.5)

				Long COVID		Long COVID (diagnoses)	
Variable	Level	*N*	Follow-up	n	Rate per 100,000 person-years (95% CI)	n	Rate per 100,000 person-years (95% CI)
No. vaccine doses	0	27,668,960	121.5	14,850	122.5 (120.5-124.5)	6,765	55.5 (54.5-57)
	1	21,205,660	37.5	5,890	156.5 (152.5-160.5)	2,335	62 (59.5-64.5)
	2	29,437,615	98.5	20,100	204.5 (201.5-207)	6,560	66.5 (65-68.5)
	3+	18,373,030	141.5	14,615	103.5 (101.5-105)	4,360	31 (30-31.5)
mRNA vaccine received	No vaccine	27,668,960	121.5	14,850	122.5 (120.5-124.5)	6,765	55.5 (54.5-57)
	non_mRNA	28,182,305	69	15,585	226.5 (223-230)	5,590	81.5 (79-83.5)
	mRNA	40,833,995	209	25,025	120 (118.5-121.5)	7,665	36.5 (36-37.5)
IMD (quintile)	1 (most deprived)	16,778,560	73.5	9,555	130 (127.5-132.5)	3,685	50 (48.5-51.5)
	2	18,155,515	76.5	10,380	135.5 (133-138.5)	3,785	49.5 (48-51)
	3	20,383,705	83.5	11,470	137.5 (135-140.5)	4,170	50 (48.5-51.5)
	4	19,637,340	79	11,840	150.5 (147.5-153)	4,055	51.5 (50-53)
	5 (least deprived)	18,619,910	73.5	10,785	147 (144-149.5)	3,770	51.5 (49.5-53)
	NA	3,110,230	13.5	1,430	106.5 (101-112)	555	41.5 (38-45)
Ethnicity	White	69,974,800	283	42,765	151 (150-152.5)	15,375	54.5 (53.5-55)
	Mixed	978,600	NA	595	133 (122.5-144)	NA	-- --
	South Asian	5,995,525	26	3,220	123 (119-127.5)	1,335	51 (48.5-54)
	Black	1,882,135	NA	885	100 (94-107)	355	40 (36-44.5)
	Other	1,932,515	NA	730	80 (74-86)	285	31.5 (28-35.5)
	NA	15,921,690	67.5	7,270	107.5 (105-110)	2,425	36 (34.5-37.5)
Comorbidities	0	59,471,410	253	30,900	122 (121-123.5)	10,935	43 (42.5-44)
	1	26,134,965	104.5	17,580	168.5 (166-171)	6,585	63 (61.5-64.5)
	2+	11,078,890	42	6,980	167 (163-170.5)	2,505	60 (57.5-62)
Shielding (high risk group)	TRUE	5,092,230	19	4,230	222 (215.5-228.5)	1,295	68 (64.5-71.5)

Table S2: Full table of all crude and adjusted rate ratios from negative binomial models of the rate of long COVID (any code), long COVID diagnosis codes, and COVID-19 hospitalisation stratified by variant period (wildtype/alpha, 1 November 2020 - 16 May 2021; Delta, 16 May 2021 - 1 December 2021; Omicron, 1 December 2021 - 31 Jan 2023) and adjusted for variant period (“All” in the “variant” column). Adjusted models are adjusted for age, sex, 9 NHS regions of England, and the dominant variant circulating.

variant	variable	level	model	long COVID	Diagnoses	Hospitalisation
0: wild/alpha	Age category	.18-29 (baseline)	adjusted	1 (1-1)	1 (1-1)	1 (1-1)
	Age category	30-39	adjusted	1.8 (1.6-2.1)	2.1 (1.7-2.5)	1.6 (1.3-1.8)
	Age category	40-49	adjusted	2.7 (2.3-3.1)	3.3 (2.8-4)	2.3 (2-2.7)
	Age category	50-59	adjusted	2.8 (2.5-3.3)	3.6 (3-4.3)	3.4 (2.9-4)
	Age category	60-69	adjusted	1.6 (1.4-1.8)	2 (1.6-2.4)	4.3 (3.7-5)
	Age category	70+	adjusted	0.69 (0.59-0.81)	0.83 (0.68-1)	7.3 (6.2-8.5)
	mRNA vaccine received	.No vaccine (baseline)	adjusted	1 (1-1)	1 (1-1)	1 (1-1)
	mRNA vaccine received	mRNA	adjusted	0.19 (0.16-0.23)	NA NA	0.24 (0.21-0.26)
	mRNA vaccine received	non_mRNA	adjusted	0.04 (0.03-0.05)	NA NA	0.19 (0.17-0.21)
	No. vaccine doses	1	adjusted	0.12 (0.1-0.14)	0.05 (0.04-0.06)	0.25 (0.23-0.27)
	No. vaccine doses	2	adjusted	NA NA	NA NA	0.04 (0.04-0.05)
	No. vaccine doses	.0 (baseline)	adjusted	1 (1-1)	1 (1-1)	1 (1-1)
	Sex	.male (baseline)	adjusted	1 (1-1)	1 (1-1)	1 (1-1)
	Sex	female	adjusted	1.8 (1.6-1.9)	1.8 (1.6-2)	1 (0.93-1.1)
	Age category	.18-29 (baseline)	crude	1 (1-1)	1 (1-1)	1 (1-1)
	Age category	30-39	crude	1.8 (1.3-2.5)	2.1 (1.5-2.9)	1.5 (1.2-1.9)
	Age category	40-49	crude	2.5 (1.8-3.5)	3.4 (2.5-4.7)	2.2 (1.8-2.8)
	Age category	50-59	crude	2.7 (1.9-3.7)	3.7 (2.7-5.1)	3.3 (2.6-4.2)
	Age category	60-69	crude	1.5 (1.1-2.1)	2 (1.4-2.8)	4.2 (3.3-5.3)
	Age category	70+	crude	0.69 (0.5-0.97)	0.88 (0.63-1.2)	7.2 (5.7-9.1)
	mRNA vaccine received	.No vaccine (baseline)	crude	1 (1-1)	1 (1-1)	1 (1-1)
	mRNA vaccine received	mRNA	crude	0.24 (0.18-0.31)	NA NA	0.25 (0.2-0.31)
	mRNA vaccine received	non_mRNA	crude	0.04 (0.03-0.06)	NA NA	0.21 (0.17-0.26)
	No. vaccine doses	1	crude	0.14 (0.11-0.18)	0.05 (0.04-0.08)	0.28 (0.23-0.35)
	No. vaccine doses	2	crude	NA NA	NA NA	0.06 (0.04-0.08)
	No. vaccine doses	.0 (baseline)	crude	1 (1-1)	1 (1-1)	1 (1-1)
	Sex	.male (baseline)	crude	1 (1-1)	1 (1-1)	1 (1-1)
	Sex	female	crude	1.7 (1.4-2.2)	1.8 (1.4-2.3)	0.86 (0.67-1.1)
1: delta	Age category	.18-29 (baseline)	adjusted	1 (1-1)	1 (1-1)	1 (1-1)
	Age category	30-39	adjusted	1.4 (1.2-1.6)	1.6 (1.4-1.8)	1.2 (1.1-1.5)
	Age category	40-49	adjusted	1.9 (1.7-2.1)	2.2 (2-2.6)	1.2 (1.1-1.5)
	Age category	50-59	adjusted	1.8 (1.6-2.1)	2.2 (2-2.6)	1.4 (1.2-1.6)
	Age category	60-69	adjusted	1.1 (0.96-1.3)	1.3 (1.1-1.5)	1.7 (1.5-2.1)
	Age category	70+	adjusted	0.43 (0.38-0.5)	0.52 (0.44-0.61)	3 (2.5-3.5)
	mRNA vaccine received	.No vaccine (baseline)	adjusted	1 (1-1)	1 (1-1)	1 (1-1)

variant	variable	level	model	long COVID	Diagnoses	Hospitalisation
	mRNA vaccine received	mRNA	adjusted	0.47 (0.43-0.52)	0.43 (0.38-0.47)	0.21 (0.19-0.23)
	mRNA vaccine received	non_mRNA	adjusted	0.72 (0.66-0.79)	0.66 (0.6-0.74)	0.32 (0.3-0.35)
	No. vaccine doses	1	adjusted	1.9 (1.7-2.2)	1.7 (1.5-2)	0.33 (0.3-0.36)
	No. vaccine doses	2	adjusted	0.42 (0.37-0.48)	0.35 (0.31-0.4)	0.25 (0.23-0.27)
	No. vaccine doses	.0 (baseline)	adjusted	1 (1-1)	1 (1-1)	1 (1-1)
	No. vaccine doses	3+	adjusted	NA NA	NA NA	0.19 (0.16-0.21)
	Sex	.male (baseline)	adjusted	1 (1-1)	1 (1-1)	1 (1-1)
	Sex	female	adjusted	1.6 (1.5-1.8)	1.8 (1.7-2)	1.1 (0.97-1.2)
	Age category	.18-29 (baseline)	crude	1 (1-1)	1 (1-1)	1 (1-1)
	Age category	30-39	crude	1.4 (1-1.9)	1.6 (1.2-2.1)	1.2 (0.95-1.5)
	Age category	40-49	crude	1.8 (1.3-2.5)	2.3 (1.7-3)	1.2 (0.92-1.5)
	Age category	50-59	crude	1.7 (1.3-2.4)	2.2 (1.7-2.9)	1.3 (1-1.6)
	Age category	60-69	crude	1 (0.74-1.4)	1.3 (0.98-1.7)	1.6 (1.3-2.1)
	Age category	70+	crude	0.44 (0.32-0.6)	0.53 (0.4-0.72)	2.9 (2.3-3.7)
	mRNA vaccine received	.No vaccine (baseline)	crude	1 (1-1)	1 (1-1)	1 (1-1)
	mRNA vaccine received	mRNA	crude	0.51 (0.42-0.61)	0.43 (0.36-0.52)	0.23 (0.19-0.28)
	mRNA vaccine received	non_mRNA	crude	0.7 (0.58-0.84)	0.63 (0.52-0.76)	0.34 (0.29-0.41)
	No. vaccine doses	1	crude	2.1 (1.7-2.6)	2.2 (1.8-2.7)	0.37 (0.3-0.44)
	No. vaccine doses	2	crude	0.46 (0.38-0.57)	0.38 (0.31-0.46)	0.28 (0.23-0.34)
	No. vaccine doses	.0 (baseline)	crude	1 (1-1)	1 (1-1)	1 (1-1)
	No. vaccine doses	3+	crude	NA NA	NA NA	0.22 (0.18-0.28)
	Sex	.male (baseline)	crude	1 (1-1)	1 (1-1)	1 (1-1)
	Sex	female	crude	1.6 (1.3-2)	1.8 (1.5-2.2)	0.97 (0.8-1.2)
2: omicron	Age category	.18-29 (baseline)	adjusted	1 (1-1)	1 (1-1)	1 (1-1)
	Age category	30-39	adjusted	1.6 (1.4-1.8)	1.8 (1.5-2)	1.1 (0.87-1.3)
	Age category	40-49	adjusted	2.1 (1.8-2.4)	2.4 (2.1-2.8)	1.1 (0.92-1.4)
	Age category	50-59	adjusted	2 (1.7-2.2)	2.3 (2-2.6)	1.6 (1.3-2)
	Age category	60-69	adjusted	1.3 (1.2-1.5)	1.5 (1.3-1.8)	2.9 (2.3-3.5)
	Age category	70+	adjusted	0.82 (0.71-0.94)	0.93 (0.8-1.1)	7.5 (6.1-9.2)
	mRNA vaccine received	.No vaccine (baseline)	adjusted	1 (1-1)	1 (1-1)	1 (1-1)
	mRNA vaccine received	mRNA	adjusted	1.3 (1.2-1.4)	1.2 (1.1-1.3)	0.73 (0.67-0.8)
	mRNA vaccine received	non_mRNA	adjusted	5.7 (5.2-6.3)	5.3 (4.8-5.9)	1.5 (1.3-1.6)
	No. vaccine doses	1	adjusted	2 (1.8-2.3)	1.9 (1.6-2.2)	1.5 (1.4-1.7)
	No. vaccine doses	2	adjusted	4.8 (4.3-5.3)	4.1 (3.6-4.6)	1.1 (1-1.2)
	No. vaccine doses	.0 (baseline)	adjusted	1 (1-1)	1 (1-1)	1 (1-1)
	No. vaccine doses	3+	adjusted	0.99 (0.89-1.1)	0.93 (0.83-1)	0.71 (0.64-0.78)
	Sex	.male (baseline)	adjusted	1 (1-1)	1 (1-1)	1 (1-1)
	Sex	female	adjusted	1.7 (1.6-1.9)	2 (1.8-2.2)	1.4 (1.2-1.6)
	Age category	.18-29 (baseline)	crude	1 (1-1)	1 (1-1)	1 (1-1)
	Age category	30-39	crude	1.6 (1.2-2.1)	1.8 (1.3-2.3)	1 (0.81-1.3)
	Age category	40-49	crude	2 (1.5-2.6)	2.4 (1.8-3.2)	1 (0.78-1.3)

variant	variable	level	model	long COVID	Diagnoses	Hospitalisation
	Age category	50-59	crude	1.9 (1.4-2.4)	2.2 (1.7-2.9)	1.4 (1.1-1.9)
	Age category	60-69	crude	1.2 (0.94-1.6)	1.5 (1.1-1.9)	2.5 (1.9-3.2)
	Age category	70+	crude	0.73 (0.55-0.96)	0.89 (0.67-1.2)	6.4 (5-8.2)
	mRNA vaccine received	.No vaccine (baseline)	crude	1 (1-1)	1 (1-1)	1 (1-1)
	mRNA vaccine received	mRNA	crude	1.2 (1-1.4)	1.1 (0.96-1.4)	0.67 (0.54-0.84)
	mRNA vaccine received	non_mRNA	crude	5.7 (4.9-6.7)	5.3 (4.4-6.3)	1.5 (1.2-1.8)
	No. vaccine doses	1	crude	1.9 (1.6-2.3)	1.8 (1.4-2.3)	1.4 (1.1-1.8)
	No. vaccine doses	2	crude	5.1 (4.3-6)	4.6 (3.8-5.6)	1.3 (1-1.6)
	No. vaccine doses	.0 (baseline)	crude	1 (1-1)	1 (1-1)	1 (1-1)
	No. vaccine doses	3+	crude	0.98 (0.83-1.2)	0.96 (0.79-1.2)	0.65 (0.51-0.82)
	Sex	.male (baseline)	crude	1 (1-1)	1 (1-1)	1 (1-1)
	Sex	female	crude	1.8 (1.5-2.1)	2 (1.7-2.4)	1.1 (0.79-1.4)
All	Age category	.18-29 (baseline)	adjusted	1 (1-1)	1 (1-1)	1 (1-1)
	Age category	30-39	adjusted	1.6 (1.4-1.8)	1.8 (1.6-2)	1.3 (1.1-1.4)
	Age category	40-49	adjusted	2.1 (1.9-2.4)	2.6 (2.3-2.8)	1.4 (1.3-1.7)
	Age category	50-59	adjusted	2.1 (1.9-2.4)	2.6 (2.3-2.9)	1.9 (1.7-2.2)
	Age category	60-69	adjusted	1.3 (1.2-1.5)	1.6 (1.4-1.8)	2.7 (2.4-3.1)
	Age category	70+	adjusted	0.66 (0.58-0.74)	0.77 (0.69-0.87)	5.5 (4.8-6.2)
	mRNA vaccine received	.No vaccine (baseline)	adjusted	1 (1-1)	1 (1-1)	1 (1-1)
	mRNA vaccine received	mRNA	adjusted	0.41 (0.36-0.47)	0.33 (0.29-0.37)	0.3 (0.28-0.32)
	mRNA vaccine received	non_mRNA	adjusted	0.87 (0.77-0.99)	0.75 (0.66-0.85)	0.45 (0.42-0.49)
	No. vaccine doses	1	adjusted	0.85 (0.73-0.99)	0.78 (0.67-0.91)	0.43 (0.39-0.47)
	No. vaccine doses	2	adjusted	0.58 (0.5-0.68)	0.53 (0.45-0.62)	0.29 (0.26-0.31)
	No. vaccine doses	.0 (baseline)	adjusted	1 (1-1)	1 (1-1)	1 (1-1)
	No. vaccine doses	3+	adjusted	0.15 (0.12-0.18)	0.16 (0.13-0.19)	0.24 (0.21-0.27)
	Sex	.male (baseline)	adjusted	1 (1-1)	1 (1-1)	1 (1-1)
	Sex	female	adjusted	1.7 (1.6-1.8)	1.8 (1.7-2)	1.1 (1.1-1.2)
	Age category	.18-29 (baseline)	crude	1 (1-1)	1 (1-1)	1 (1-1)
	Age category	30-39	crude	1.5 (1.2-1.9)	1.7 (1.4-2.1)	1.2 (1-1.5)
	Age category	40-49	crude	2 (1.6-2.5)	2.6 (2.1-3.1)	1.4 (1.2-1.6)
	Age category	50-59	crude	1.9 (1.6-2.4)	2.5 (2.1-3)	1.9 (1.6-2.2)
	Age category	60-69	crude	1.2 (0.94-1.4)	1.5 (1.2-1.8)	2.7 (2.2-3.1)
	Age category	70+	crude	0.59 (0.47-0.73)	0.71 (0.59-0.87)	5.4 (4.6-6.4)
	mRNA vaccine received	.No vaccine (baseline)	crude	1 (1-1)	1 (1-1)	1 (1-1)
	mRNA vaccine received	mRNA	crude	0.62 (0.52-0.74)	0.48 (0.41-0.57)	0.37 (0.32-0.43)
	mRNA vaccine received	non_mRNA	crude	1.6 (1.3-1.9)	1.1 (0.97-1.3)	0.61 (0.53-0.7)
	No. vaccine doses	1	crude	1.6 (1.3-2)	1.5 (1.2-1.8)	0.62 (0.53-0.73)
	No. vaccine doses	2	crude	1.4 (1.1-1.7)	0.99 (0.82-1.2)	0.5 (0.43-0.58)
	No. vaccine doses	.0 (baseline)	crude	1 (1-1)	1 (1-1)	1 (1-1)
	No. vaccine doses	3+	crude	0.38 (0.31-0.48)	0.31 (0.25-0.39)	0.42 (0.35-0.49)
	Sex	.male (baseline)	crude	1 (1-1)	1 (1-1)	1 (1-1)

variant	variable	level	model	long COVID	Diagnoses	Hospitalisation
	Sex	female	crude	1.7 (1.5-1.9)	1.9 (1.6-2.1)	0.96 (0.82-1.1)

Table S3: Demographic and clinical characteristics at baseline of 55,465 individuals with a record of long COVID during the study period, stratified by whether they had a record of a positive SARS-COV-2 test at least 12 weeks before the long COVID code. Evidence for a difference between those with/without a positive test are shown with *p*-values from a Chi-squared test for each "Variable". All counts <10 have been redacted and rounded to the nearest 5

**Variable**	**Level**	**No positive test**	**Test positive**	***p***
Diagnosis or referral code	Dx	9080 (27.8)	4930 (21.6)	p<0.001
	Rx	23605 (72.2)	17845 (78.4)	
Sex	male	12360 (37.8)	7730 (33.9)	p<0.001
	female	20325 (62.2)	15050 (66.1)	
Age category	18-29	4700 (14.4)	2885 (12.7)	p<0.001
	30-39	6340 (19.4)	4305 (18.9)	
	40-49	7620 (23.3)	5975 (26.2)	
	50-59	7595 (23.2)	5975 (26.2)	
	60-69	3820 (11.7)	2530 (11.1)	
	70+	2610 (8)	1105 (4.9)	
Region	London	1560 (4.8)	1085 (4.8)	p<0.001
	East Midlands	3845 (11.8)	3965 (17.4)	
	East	6350 (19.4)	4360 (19.2)	
	North East	2415 (7.4)	1865 (8.2)	
	North West	3815 (11.7)	2500 (11)	
	South East	2155 (6.6)	1695 (7.4)	
	South West	5925 (18.1)	2735 (12)	
	West Midlands	1025 (3.1)	740 (3.3)	
	Yorkshire and The Humber	5585 (17.1)	3815 (16.8)	
Ethnicity	White	25390 (89.6)	17375 (87.6)	p<0.001
	Mixed	365 (1.3)	230 (1.2)	
	South Asian	1635 (5.8)	1585 (8)	
	Black	505 (1.8)	380 (1.9)	
	Other	450 (1.6)	275 (1.4)	
IMD (quintile)	1 (most deprived)	5100 (16)	4455 (20.1)	p<0.001
	2	5865 (18.4)	4515 (20.3)	
	3	6685 (21)	4790 (21.6)	
	4	7390 (23.2)	4450 (20.1)	
	5 (least deprived)	6805 (21.4)	3980 (17.9)	
# comorbidities	0	18245 (55.8)	12660 (55.6)	0.29
	1	10285 (31.5)	7295 (32)	
	2+	4155 (12.7)	2825 (12.4)	
# vaccines	0	8940 (27.4)	5900 (25.9)	p<0.001

Variable	Level	No positive test	Test positive	*p*
	1	2745 (8.4)	2935 (12.9)	
	2	12390 (37.9)	7325 (32.2)	
	3+	8615 (26.4)	6615 (29)	
# positive tests	0	32685 (100)		p<0.001
	1		15690 (68.9)	
	2		4670 (20.5)	
	3		1275 (5.6)	
	4		495 (2.2)	
	5+		645 (2.8)	
# COVID-19 hospitalisations	0	32410 (99.2)	19420 (85.3)	p<0.001
	1	265 (0.8)	2950 (13)	
	2	10 (0)	330 (1.4)	
	3+	NA	75 (0.3)	

Table S3: Items That Should Be Included in Reports of Descriptive Studies (https://doi.org/10.1093/aje/kwac115)

Article Section and Item	No.	Recommendation(s)	Section where recommendation is addressed
Title and abstract	1	Explicitly state that this is a “descriptive study” in the title or the abstract.	Abstract: discussion
	2	Summarize the target population and provide an informative and balanced summary of estimated disease occurrence in the abstract.	Abstract
Introduction			
Background/rationale	3	State the motivation for the study, including, where relevant, the action that might be informed by the results.	Background
Objectives	4	State the descriptive estimand, explicitly including:<br>(a) the target population (who would be affected by any decisions made as a result of the study?);<br>(b) the health state to be summarized;<br>(c) the measure of occurrence; and<br>(d) any stratification variables, if applicable.	Background (last sentence)
Methods			
Study design	5	(a) State whether the study is cross-sectional or longitudinal.<br>(b) Restate the measure of occurrence being targeted.<br>(c) If the study is longitudinal, specify the time origin and follow-up period for the measure of occurrence; if the study is cross-sectional, specify the time anchor at which the health state is summarized for individuals.	a) Referred to as “cohort” throughout<br>b) Primary analysis<br>c) Study Population

Setting	6	Describe any relevant features of the place and time in which the target population resides and across which data were collected.	Data Source and Study Population
Participants	7	(a) Describe the target population thoroughly in terms of person, place, and time.<br>(b) Describe sampling into the study population (whether sampling was explicit or implicit, e.g., by inclusion in an administrative database); this includes eligibility criteria (see recommendations on data sources in item 10 below).<br>(c) Describe any restrictions on the analytical sample.	Data Source and Study Population
Outcome(s)	8	(a) State when and how the outcome is measured.<br>(b) Include estimates or discussion of the sensitivity and specificity of the study outcome definition relative to the gold standard.<br>(c) List secondary outcomes or competing events of interest.	Outcomes
Covariates	9	Specify any stratification or adjustment variables—clearly define how variables were collected or constructed.	Stratifiers
Data sources/measurement	10	Clearly delineate any inclusion/exclusion criteria for membership in the data source, including the original purpose for which the data were collected, if not for the study at hand.	Data Source
Bias	11	Describe any assumptions or methods used to extrapolate data from the analytical sample to the study population and from the study population to the target population.	Discussion
Statistical methods	12	(a) Describe the primary statistical methods used to estimate the measure of disease occurrence being targeted; discuss assumptions of that method in light of data limitations (e.g., assumption of independent censoring for people lost to follow-up).<br>(b) If any adjustment/standardization will be done, state the goal of such adjustment.	Statistical methods
Results			
Participants	13	Report numbers of individuals at each study stage (this is likely to be approximate for the target population); consider summarizing this information in a flow diagram.	Variation in incidence of long COVID recording in England & Table 2
Descriptive data	14	(a) Report on the characteristics of the analytical sample in a “Table 1.”<br>(b) Indicate the number of participants with missing data for each variable used in the analysis.<br>(c) If any weighting or imputation is done to reconstruct the study sample or target populations, include columns for those populations.	Table 2
Outcome data	15	(a) Present an overall (unstratified) estimate of the measure of occurrence of interest.<br>(b) Report “crude” (raw data in the analytical sample) and (if applicable) “corrected” (after any weighting or imputation) estimates.	Recorded long COVID rates vary between population groups & Figure 3
Other analyses	16	Present prespecified stratum-specific or adjusted/standardized results.	Figure 3 and Figure S7

Discussion			
Key results	17	Summarize key results with reference to the study objectives.	Discussion
Limitations	18	Summarize potential sources of selection bias and measurement error and any attempts to mitigate these biases. Discuss both the direction and magnitude of any potential bias. Integrating quantitative bias analysis into the study to guide these discussions is encouraged.	Discussion
Interpretation	19	(a) Avoid causal interpretations of descriptive results; avoid overinterpreting stratum-specific differences in measures of occurrence.<br>(b) Describe how results of this study might inform or improve public health or clinical practice.	Discussion
