# Supplementary material for: Clinical coding of long COVID in primary care 2020–2023 in a cohort of 19 million adults: an OpenSAFELY analysis
Source: eClinicalMedicine. 2024 May 17;72:102638. doi: 10.1016/j.eclinm.2024.102638 (PMC11127160; doi:10.1016/j.eclinm.2024.102638)
Supplement: OS-collaborative list [file mmc2.pdf]

First name	Last name
Alex	Walker
Amelia	Green
Amir	Mehrkar
Andrea	Schaffer
Andrew	Brown
Ben	Goldacre
Ben	Butler-Cole
Brian	MacKenna
Caroline	Morton
Caroline	Walters
Catherine	Stables
Christine	Cunningham
Christopher	Wood
Colm	Andrews
David	Evans
George	Hickman
Helen	Curtis
Henry	Drysdale
Iain	Dillingham
Jessica	Morley
Jon	Massey
Linda	Nab
Lisa	Hopcroft
Louis	Fisher
Lucy	Bridges
Milan	Wiedemann
Nicholas	DeVito
Orla	Macdonald
Peter	Inglesby
Rebecca	Smith
Richard	Croker

Robin	Park
Rose	Higgins
Sebastian	Bacon
Simon	Davy
Steven	Maude
Thomas	O'Dwyer
Tom	Ward
Victoria	Speed
William	Hulme
Liam	Hart
Pete	Stokes
Krishnan	Bhaskaran
Ruth	Costello
Thomas	Cowling
Ian	Douglas
Rosalind	Eggo
Stephen	Evans
Harriet	Forbes
Richard	Grieve
Daniel	Grint
Emily	Herrett
Sinead	Langan
Viyaasan	Mahalingasivam
Kathryn	Mansfield
Rohini	Mathur
Helen	McDonald
Edward	Parker
Christopher	Rentsch
Anna	Schultze
Liam	Smeeth
John	Tazare
Laurie	Tomlinson
Jemma	Walker

Elizabeth	Williamson
Kevin	Wing
Angel	Wong
Bang	Zheng
Christopher	Bates
Jonathan	Cockburn
John	Parry
Frank	Hester
Sam	Harper
Shaun	O'Hanlon
Alex	Eavis
Richard	Jarvis
Dima	Avramov
Paul	Griffiths
Aaron	Fowles
Nasreen	Parkes
Rafael	Perera
David	Harrison
Kamlesh	Khunti
Jonathan	Sterne
Jennifer	Quint
